# Treatment outcomes of extensively drug-resistant tuberculosis in Europe: a retrospective cohort study

**DOI:** 10.1016/j.lanepe.2025.101380

**Published:** 2025-07-15

**Authors:** Yousra Kherabi, Ole Skouvig Pedersen, Christoph Lange, François Bénézit, Dumitru Chesov, Luigi Ruffo Codecasa, Andrii Dudnyk, Nana Kiria, Olha Konstantynovska, Dhiba Marigot-Outtandy, Traian-Constantin Panciu, Corentin Poignon, Sirje Sasi, Dagmar Schaub, Varvara Solodovnikova, Laima Vasiliauskaitè, Lusine Yeghiazaryan, Gunar Günther, Lorenzo Guglielmetti, TBnet/ESGMYC XDR-TB Study Groupag, Alexandra Aubry, Anca Vasiliu, Andrii Dudnyk, Anne Marie McLaughlin, Christoph Lange, Corentin Poignon, Dagmar Schaub, Daniela Cirillo, Daria Podlekareva, Dhiba Marigot-Outtandy, Dumitru Chesov, Edita Davidavičienė, François Bénézit, Giovanni Fumagalli, Giuliana Troia, Gunar Günther, Ilaria Motta, Jerker Jonsson, Jérôme Robert, Johannes Eimer, Laima Vasiliauskaitè, Liga Kuska, Lorenzo Guglielmetti, Luigi Ruffo Codecasa, Lusine Yeghiazaryan, Marcin Skowroński, Margaret Fitzgibbon, Mathieu Revest, Nana Kiria, Nicolas Veziris, Ole Skouvig Pedersen, Olha Konstantynovska, Onya Opota, Piret Viiklepp, Roxana Coriu, Simone Tunesi, Sirje Sasi, Sten Skogmar, Stephanie Bjerrum, Tamar Togonidze, Traian-Constantin Panciu, Troels Lillebaek, Valérie Pourcher, Varvara Solodovnikova, Vivian Bui Le, Yousra Kherabi

**Affiliations:** a Infectious and Tropical Diseases Department, Bichat-Claude Bernard Hospital, Assistance Publique-Hôpitaux de Paris, Université Paris Cité, Paris, France; b Université Paris Cité, Inserm, IAME, Paris, France; c Department of Respiratory Medicine and Allergy, Aarhus University Hospital, Aarhus, Denmark; d Department of Clinical Medicine, Aarhus University, Aarhus, Denmark; e Division of Clinical Infectious Diseases, Research Center Borstel, Borstel, Germany; f German Center for Infection Research (DZIF), Partner Site Hamburg-Lübeck-Borstel-Riems, Borstel, Germany; g Respiratory Medicine and International Health, University of Lübeck, Lübeck, Germany; h Baylor College of Medicine and Texas Children Hospital, Global TB Program, Houston, TX, USA; i Infectious Disease and Intensive Care Unit, Pontchaillou University Hospital, Rennes, France; j Nicolae Testemitanu State University of Medicine and Pharmacy, Chisinau, Republic of Moldova; k Chiril Draganiuc Pneumology Institute, Chisinau, Republic of Moldova; l Regional TB Reference Centre, Instituto Villa Marelli, ASST Grande Ospedale Metropolitano Niguarda, Milan, Italy; m Department of Tuberculosis, Clinical Immunology and Allergy, National Pirogov Memorial Medical University, Vinnytsia, Ukraine; n Institut d'Investigació Germans Trias i Pujol (IGTP), Barcelona, Spain; o National Center for Tuberculosis and Lung Diseases, Tbilisi, Georgia; p Department of Infectious Diseases and Clinical Immunology, V.N. Karazin Kharkiv National University, Kharkiv, Ukraine; q “Regional Phtisiopulmonological Center” of the Kharkiv Regional Council, Communal Non-commercial Enterprise, Kharkiv, Ukraine; r Department of Infectious Disease, Imperial College London, London, UK; s Sanatorium, Centre Hospitalier de Bligny, Briis-sous-Forges, France; t Marius Nasta Institute of Pneumology Bucharest, Bucharest, Romania; u Sorbonne-Université, INSERM, CNRS, Centre d’Immunologie et de Maladies Infectieuses, CIMI, Paris, France; v Assistance Publique Hôpitaux de Paris, Groupe Hospitalier Universitaire Sorbonne Université, Hôpital Pitié-Salpêtrière, Centre National de Référence des Mycobactéries et de la Résistance des Mycobactéries aux Antituberculeux, Paris, France; w Department of Microbiology and Molecular Diagnostics, Laboratory, North Estonia Medical Centre, Tallinn, Estonia; x TBnet, Bad Oldesloe, Germany; y Department of Physiology, Biochemistry, Microbiology and Laboratory Medicine, Institute of Biomedical Sciences, Faculty of Medicine, Vilnius University, Vilnius, Lithuania; z Centre of Laboratory Medicine, Laboratory of Infectious Diseases and Tuberculosis, Vilnius University Hospital Santaros Klinikos, Vilnius, Lithuania; aa National Center of Pulmonology, Abovyan, Republic of Armenia; ab Department of Pulmonary Medicine, Allergology and Clinical Immunology, Inselspital, Bern University Hospital, University of Bern, Bern, Switzerland; ac Department of Medical Sciences, School of Medicine, University of Namibia, Windhoek, Namibia; ad Department of Infectious, Tropical Diseases and Microbiology, IRCCS Sacro Cuore Don Calabria Hospital, Negrar di Valpolicella, Verona, Italy

**Keywords:** TB, TBnet, MDR/RR-TB, Pre-XDR-TB, XDR-TB, ESGMYC

## Abstract

**Background:**

In 2021, World Health Organization revised of definition of extensive drug-resistant tuberculosis. We aimed to determine treatment outcomes of individuals affected by extensively drug-resistant tuberculosis in Europe.

**Methods:**

This observational, retrospective cohort study included patients diagnosed with extensively drug-resistant tuberculosis in the World Health Organization European Region from 2017 to 2023. Participating centres collected consecutive, detailed individual data for extensively drug-resistant tuberculosis patients. Data were analysed with meta- and regression methods, accounting for between-country heterogeneity.

**Findings:**

Among 11,003 patients with multidrug-resistant/rifampicin-resistant tuberculosis, 188 (1·7%) from 16 countries had extensively drug-resistant tuberculosis. Of these, 48·4% harboured strains with resistance to bedaquiline (n = 91/188), 34·0% to linezolid (n = 64/188), and 17·6% to both (n = 33/188). The individual composition of anti-tuberculosis regimens was highly variable, with 151 different drug combinations. Among the 156/188 (83·0%) patients with available treatment outcomes, the pooled percentage of successful outcomes was 40·2% (95% confidence interval [95% CI] 28·4%–53·2%). In patients with unsuccessful treatment outcomes (101/156), most experienced treatment failure (n = 57/156 [pooled proportion 37·1%], 95% CI: 26·1%–49·7%) or death (n = 30/156 [pooled proportion 21·3%], 95% CI: 15·7%–28·2%). After adjustment for disease severity, each additional likely effective drug decreased the odds of unsuccessful outcomes (adjusted odds ratio: 0·65, 95% CI: 0·45–0·96) (p = 0·026), whereas being treated in an upper-middle-income country increased the odds of unsuccessful outcomes compared with being treated in a high-income country (adjusted odds ratio: 13·7, 95% CI: 3·7–50·2) (p < 0·001). Compared with other levels of drug resistance, treatment outcomes were significantly worse for extensively drug-resistant tuberculosis.

**Interpretation:**

Only four out of ten patients affected by extensively drug-resistant tuberculosis achieved successful treatment outcomes. These findings highlight the need for adequate, individualised treatment regimens and optimised drug susceptibility testing.

**Funding:**

None.

**Research in context**

**Evidence before this study**

Rifampicin-resistant tuberculosis is one of four organisms with critical priority in the World Health Organization (WHO) bacterial priority pathogen list. In 2021, based on the analysis of a large individual patient data database, the WHO revised its definitions of drug-resistant tuberculosis. Pre-extensively drug-resistant tuberculosis is now defined as disease due to *Mycobacterium tuberculosis* resistant to rifampicin (with or without isoniazid resistance, i.e., multidrug-resistant/rifampicin-resistant tuberculosis) and to any fluoroquinolone, and extensively drug-resistant tuberculosis as pre-extensively drug-resistant tuberculosis plus resistance to at least one Group A drug (bedaquiline and/or linezolid). According to WHO estimates, the global treatment success rate in 2023 was 88% for drug-susceptible tuberculosis, in stark contrast with only 63% for patients with multidrug-resistant/rifampicin-resistant tuberculosis. Concerningly, treatment outcomes for patients affected by the revised form of extensively drug-resistant tuberculosis remain largely unknown. To assess available evidence, we searched PubMed on June 18, 2025, using the terms (“extensively drug-resistant” OR “XDR”) AND (“tuberculosis” OR “mycobacterium tuberculosis” OR “TB” OR “MTB” OR “tuberculosis”[Mesh]) AND “outcome∗”, restricted to peer-reviewed articles published after the 2021 definition revision. Of 226 results, only two studies included patients with the revised form of extensively drug-resistant tuberculosis. One study from France included nine such patients but did not report their outcomes separately from pre-extensively drug-resistant tuberculosis patients. Another study from five Eastern European countries (Georgia, Kazakhstan, Kyrgyzstan, Moldova, and Ukraine) included 52 patients with extensively drug-resistant tuberculosis and reported a success rate of 31%. To our knowledge, no published study has systematically described how these patients are managed. In addition, existing data on outcomes are limited by small sample sizes or restricted to smaller regions of Europe.

**Added value of this study**

This study used individual patient-level data to describe management and treatment outcomes of patients with extensively drug-resistant tuberculosis across the WHO European Region. Among 11,003 multidrug-resistant/rifampicin-resistant tuberculosis patients who received care between 2017 and 2023 in TB treatment centres in 16 countries of the WHO European Region, 188 patients fulfilled criteria for extensively drug-resistant tuberculosis. In a pooled analysis, accounting for between-country heterogeneity, only 40% of these patients achieved successful treatment outcomes, a significantly lower success rate compared to other forms of drug-resistant tuberculosis, and comparable to rates of long-term success of the pre-antibiotic era. We identified key risk factors for unsuccessful outcomes, such as the number of effective drugs in the regimen and being treated in upper middle-income countries compared with high-income countries.

**Implications of all the available evidence**

With currently available diagnostics, drugs, and infrastructures, the prognosis of patients affected by extensively drug-resistant tuberculosis in the WHO European Region is extremely poor, in upper middle-income countries. On average, half of the drugs that patients with extensively drug-resistant tuberculosis received were ineffective according to drug susceptibility testing. Findings of this study underscore the urgent need for access to rapid drug susceptibility testing for all drugs in use and effective treatment regimens, including new compounds, for all patients affected by extensively drug-resistant tuberculosis and implementation of a global antibiotic stewardship program to address growing *M tuberculosis* drug resistance. Future public health efforts should focus not only on research and development but also on implementation to improve outcomes for individuals with drug-resistant tuberculosis globally.

## Introduction

Tuberculosis remains a major global health challenge, particularly with the emergence of drug-resistant forms of the disease. In 2024, the World Health Organization (WHO) ranked multidrug-resistant/rifampicin-resistant tuberculosis, together with carbapenem-resistant *Acinetobacter baumannii*, third-generation cephalosporine-resistant Enterobacteriales and carbapenem-resistant Enterobacteriales, as the top priority bacterial pathogens globally.^1^ Multiple reports suggest that resistance to both new and repurposed anti-tuberculosis drugs is emerging worldwide.^2^, ^3^, ^4^ The case of bedaquiline is emblematic, as both primary and acquired drug resistance to this drug are increasing.^5^, ^6^, ^7^, ^8^ In the WHO European region, 35·1% of pulmonary multidrug-resistant/rifampicin-resistant tuberculosis cases had fluoroquinolone resistance (pre-extensively drug-resistant tuberculosis) in 2022.^9^ Among patients tested for any other Group A drug, the prevalence of extensively drug-resistant tuberculosis defined, according to the new 2021 WHO definitions, as pre-extensively drug-resistant tuberculosis with additional resistance to at least one additional Group A drug (either bedaquiline or linezolid),^10^ was 8·7%. However, these figures should be interpreted with caution due to the limited drug susceptibility testing (DST) capacity for bedaquiline and linezolid. Both genotypic and phenotypic DST availability is concerningly inadequate worldwide, including in the WHO European region.^11^

In response to the evolving drug resistance landscape, the WHO released new guidelines in 2022 recommending a 6-month regimen containing bedaquiline, linezolid, pretomanid and moxifloxacin (BPaLM) for multidrug-resistant/rifampicin-resistant tuberculosis treatment.^12^ For pre-extensively drug-resistant tuberculosis, the WHO endorses the same regimen excluding moxifloxacin (BPaL). In April 2025, the WHO consolidated its guidelines recommending four additional 6- and 9-month regimens, which have the advantage of being indicated also for children, pregnant and breastfeeding women.^13^ However, for extensively drug-resistant tuberculosis there are currently no alternatives to the long (18–24 months) individualised treatment regimens with poor evidence base, which often involve multiple drugs, including injectable agents, and are associated with significant toxicity. Treatment recommendations for the treatment of extensively drug-resistant tuberculosis are lacking, apart from a recently published expert consensus document.^14^

The 2021 WHO revision of drug-resistance definitions was based on a large meta-analysis of individual patient data showing that outcomes for patients treated without fluoroquinolones and bedaquiline/linezolid were significantly worse than for other multidrug-resistant/rifampicin-resistant tuberculosis patients.^15^ Emerging evidence suggests that bedaquiline resistance is associated with worse treatment outcomes.^16^ However, despite growing concerns, to date there is a notable lack of publicly available data on treatment outcomes of newly-defined extensively drug-resistant tuberculosis cases.

This study aims to evaluate the treatment approaches and outcomes of extensively drug-resistant tuberculosis patients in the WHO European region, addressing the critical gap in knowledge and informing future tuberculosis management strategies. To our knowledge, this is the largest report of its kind to date in the WHO European region.

## Methods

### Study design and data collection

In this observational, retrospective cohort study, participation was offered through the contact lists of members of the Tuberculosis Network European Trials group^17^ (TBnet; www.tbnet.eu) and the European Society of Clinical Microbiology and Infectious Diseases (ESCMID; www.escmid.com) Study Group for Mycobacteria (ESGMYC) in countries of the WHO European Region during the fourth quarter of 2023. In total, 42 centres were invited to participate. The electronic case report form (eCRF) was distributed to interested centres in the first quarter of 2024 and retrieved by July 31st 2024. Data were recorded passively by participating clinicians through retrospective review of medical charts. The study was reported in accordance with the STROBE guideline for cohort studies (Table S1 in Supplementary Material).

### Population and definitions

All participating centres treated at least one extensively drug-resistant tuberculosis patient between January 2017 and December 2023. The study population encompassed aggregate data on all multidrug-resistant/rifampicin-resistant tuberculosis cases treated during this period, with individual patient-level data specifically collected for extensively drug-resistant tuberculosis cases as per the 2021 WHO definitions.^10^ Data collected included patient characteristics, tuberculosis localisation and extent, previous tuberculosis treatment, extended phenotypic and genotypic DST results, treatment regimens and changes, treatment duration, treatment outcomes as reported by the participating centres (per WHO 2021 criteria^18^) (Table S2), post-treatment follow-up and time to sputum culture conversion (see Appendix for full eCRF).

Regarding treatment outcomes, only the WHO-defined outcome itself was reported, without further specification of the underlying reason. As such, ‘treatment failure’ may reflect either microbiological failure or clinical reasons, including regimen changes. Notably, ‘lost to follow-up’ refers to a patient whose treatment was interrupted for two or more consecutive months, whereas those with missing outcome data were classified as ‘not evaluated’. Lost to follow-up was considered an unsuccessful outcome as there was sufficient information to discriminate these patients from those who were entirely not evaluated.

Centre-level data included the number of multidrug-resistant/rifampicin-resistant tuberculosis, pre-extensively drug-resistant tuberculosis, and extensively drug-resistant tuberculosis cases and aggregate proportions of favourable outcomes for multidrug-resistant/rifampicin-resistant tuberculosis and pre-extensively drug-resistant tuberculosis during the study period.

Drugs were considered part of the treatment regimen if they were administered for at least 30 days. Resistance was defined by any resistant result on phenotypic and genotypic DST, with a conservative, clinically based approach, in which treatment regimens are typically designed according to a “worst-case scenario” regarding drug resistance. A drug was considered ‘likely effective’ in case of a proven susceptible genotypic and/or phenotypic DST result and in absence of any resistant result on genotypic and/or phenotypic DST. We categorised participating countries into high-income countries (HIC) and upper middle-income countries (UMIC) according to the 2024 World Bank classification.^19^

### Statistical analyses

Demographics and clinical characteristics were summarised for all patients using descriptive statistics. Continuous variables were presented as medians and interquartile ranges (IQRs), and categorical variables were presented as proportions. To account for heterogeneity between participating countries, we conducted a meta-analysis to estimate pooled percentages of successful outcomes and each of the individual unsuccessful outcomes, stratified by country and type of resistance (multidrug-resistant/rifampicin-resistant, pre-extensively drug-resistant, or extensively drug-resistant tuberculosis). Between-country variance was estimated using the DerSimonian-Laird estimator (τ^2^), and overall heterogeneity was expressed as the I^2^ statistic (using the R package *meta*). Proportions were logit-transformed to stabilise variances, and inverse variance weighting was applied. Clopper–Pearson confidence intervals were calculated for individual study estimates, and Knapp–Hartung adjustments were used for the random-effects models. Subgroup differences were assessed using a chi-squared test, comparing the pooled percentages between resistance groups. Patients who were ‘not evaluated’ were excluded from outcome analyses. To assess potential selection bias from excluding these patients, their baseline socio-demographic and clinical characteristics were compared to the analysed population. In a subgroup analysis, pooled individual outcomes (‘cured’, ‘treatment completed’, ‘treatment failure’, ‘died’, and ‘lost to follow-up’) were compared by subgroups based on country income level, number of likely effective anti-tuberculosis drugs included in the regimen, and resistance pattern (resistance to linezolid, bedaquiline, or both drugs). In another subgroup analysis, demographic and clinical characteristics were compared between patients with and without evidence of bedaquiline resistance who nevertheless received bedaquiline. In addition, the pooled proportion of successful outcomes was compared between patients with evidence of bedaquiline resistance who received bedaquiline and all other patients who either did not receive bedaquiline or did not have evidence of bedaquiline resistance. In a regression analysis, unadjusted odds ratios (ORs) were calculated to estimate the association between unsuccessful outcomes and four *a priori* selected covariates of interest: income category of the country where the patients were treated, resistance pattern (resistance to bedaquiline, linezolid, or both drugs), the number of total anti-tuberculosis drugs in the regimen, and the number of likely effective anti-tuberculosis drugs in the regimen. The latter two were treated as continuous variables, and the assumption of linearity between the log odds and these variables was assessed visually by plotting the fitted regression lines alongside the observed log-odds (with 95% confidence intervals) for each distinct value (Figures S1 and S2 in Supplementary Material). Adjusted odds ratios (aORs) were calculated using multivariable logistic regression models. Due to sample size constraints, four separate models were constructed, each including one of the four covariates of interest described above, along with three covariates related to disease severity that were also selected *a priori*: presence of lung cavities, sputum smear positivity, and prior treatment with second-line injectable drugs. The regression analyses were performed using complete case analysis, excluding patients with missing data. To account for potential clustering of outcomes within countries, 95% confidence intervals were calculated using cluster-robust standard errors, based on the sandwich estimator from the *sandwich* and *lmtest* R packages, with clustering by country. In the main analyses, drugs that were administered without available DST results were considered as likely non-effective, reflecting that lack of evidence of susceptibility limits confidence in their efficacy. In a sensitivity analysis, we revised this definition to classify administered drugs with missing DST results as likely effective, reflecting a more programmatic approach where drugs are often used despite the absence of DST results. In another sensitivity analysis, drugs without DST in more than 40% of patients were considered ineffective for all patients. To assess the risk of selection bias, descriptive characteristics of excluded patients were compared with the included patients.

The risks of unsuccessful outcome and death overtime were examined using Kaplan–Meier curves and stratified according to income category and the number of likely effective drugs; strata were compared using the log-rank test. Using the same variables and approach as the logistic regression, univariable and multivariable adjusted hazard ratios were estimated based on Cox regression analysis using the same covariates as the logistic regression (Figure S3 in Supplementary Material). Time at risk was defined as the interval from the reported start of treatment to the assignment of a treatment outcome. Because loss to follow-up was classified as an unsuccessful treatment outcome, these cases were not censored, whereas patients with unevaluated outcomes were excluded entirely.

Data were analysed using R version 4·2·3.

### Ethical considerations

The Institutional Review Board of the Bligny Hospital (Briis-sous-Forges, France) granted ethical clearance for this study (CRE 27/06/2023). Individual consent was waived based on anonymous data collection. Additional ethical approval was obtained in each participating centre, as needed.

### Role of the funding source

The German Center of Infection Research (DZIF) supports C.L. under Grant Agreement 02.702 for clinical tuberculosis research. DZIF had no role in the study concept, evaluation or in the decision to submit the manuscript for publication.

## Results

**Fig. 1:**
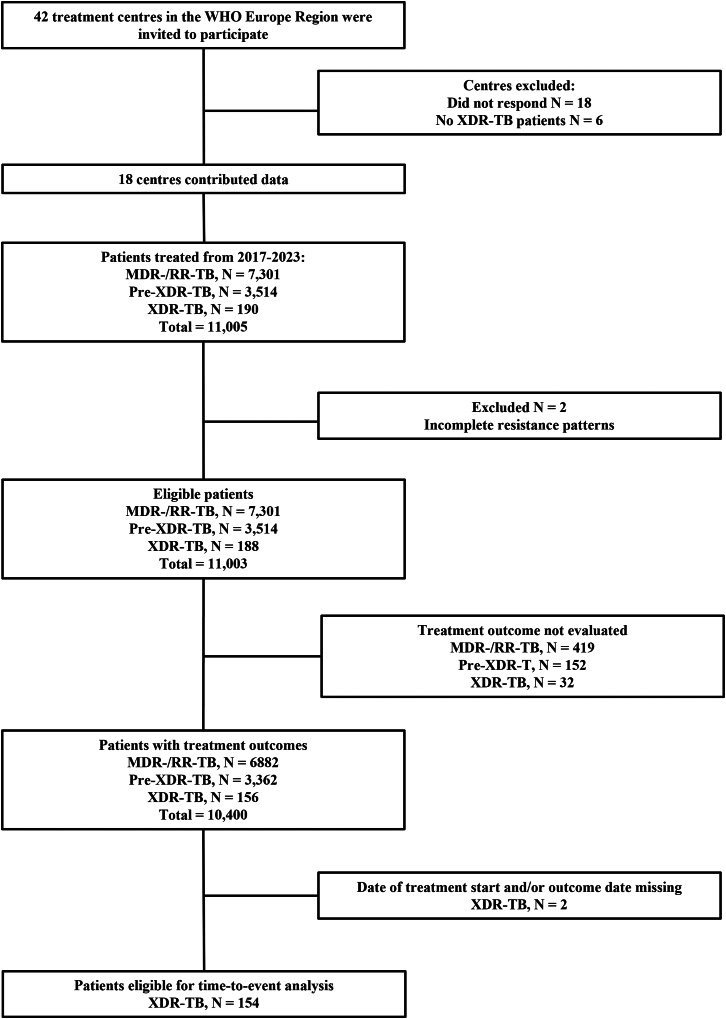
**Flow diagram.** Abbreviations: MDR-/RR-TB, multidrug/rifampicin-resistant tuberculosis; Pre-XDR-TB, pre-extensively drug-resistant tuberculosis; XDR-TB, extensively drug-resistant tuberculosis.

**Fig. 2:**
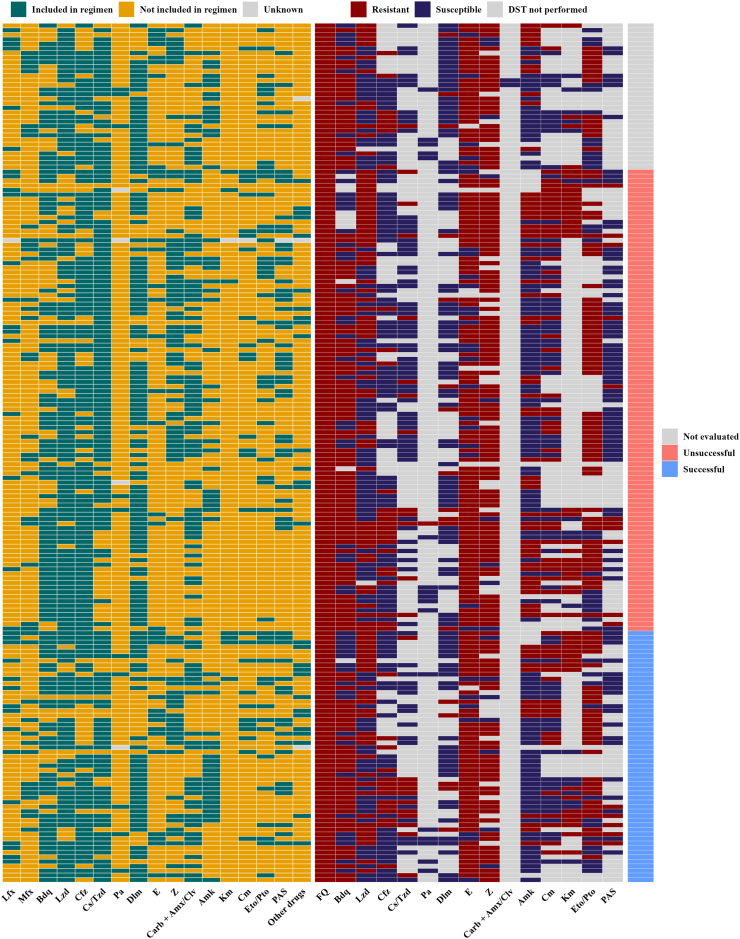
**Overview of individual treatment regimens, *Mycobacterium tuberculosis* drug susceptibility patterns, and treatment outcomes for 188 extensively drug-resistant tuberculosis patients from 16 countries in WHO Europe Region (2017–2023).** Individual-level visualisation of treatment regimens, drug susceptibility testing (DST) results, and outcomes for patients with drug-resistant TB. Each row represents one patient. The left section indicates whether specific drugs were included in the treatment regimen (green), not included (orange), or unknown (grey). The middle section shows DST results for the same drugs: resistant (dark red), susceptible (purple), or not performed (light grey). The right section displays treatment outcomes: successful (blue), unsuccessful (red), or not evaluated (light grey). Abbreviations: FQ, fluoroquinolones; Lfx, levofloxacin; Mfx, moxifloxacin; Bdq, bedaquiline; Lzd, linezolid; Cfz, clofazimine; Cs/Tzd, cycloserine/terizidone; E, ethambutol; Dlm, delamanid; Pa, pretomanid; Z, pyrazinamide; Carb + Amx/Clav, carbapenem + amoxicillin/clavulanic acid; Amk, amikacin; Cm, capreomycin; Km, kanamycin; Eto/Pto, ethionamide/prothionamide; PAS, P-aminosaliscylic acid.

Of the 42 centres invited to participate, 24 responded and 18 completed the eCRF, providing aggregate data for 11,003 patients—of whom 7301 had multidrug-resistant/rifampicin-resistant tuberculosis and 3514 had pre-extensively drug-resistant tuberculosis—as well as individual-level data for 188 patients with extensively drug-resistant tuberculosis (Fig. 1). The patients were included from 18 centres in 16 different countries (Figure S4 in Supplementary Material). Comorbidities, socio-demographic, and clinical characteristics of extensively drug-resistant tuberculosis patients are available in Table 1, Table 2. The median treatment duration was 382·0 days (IQR 242·0, 567·0), and the regimens included a median of six anti-tuberculosis drugs (IQR 5, 8), out of which a median of three were considered effective (IQR 2, 4). The individual composition of anti-tuberculosis regimens was highly variable, with 151 different drug combinations. Likewise, the individual drug-resistance patterns were diverse, including 105 distinct combinations (Fig. 2). The most frequently administered drug was linezolid (80·3%, n = 151/188) followed by cycloserine/terizidone (78·2%, n = 147/188), clofazimine (75·5%, n = 142/188), and delamanid (72·9%, n = 137/188) (Figure S5A). Significant differences were observed in the treatment regimens used in high-income countries (HICs) compared to upper-middle-income countries (UMICs) with patients in HICs receiving significantly more drugs (median: 7 vs 6, p = 0·007) and more frequently receiving moxifloxacin, pretomanid, and amikacin (Table S3 in Supplementary Material). Overall, among group A drugs, in 48·4% of patients *M tuberculosis* strains were resistant to bedaquiline alone (n = 91/188), in 34·0% to linezolid alone (n = 64/188), and in 17·6% to both drugs (n = 33/188). In addition to group A drugs, resistance was most reported to ethambutol (80·3%, n = 151/188) followed by pyrazinamide (73·4%, n = 138/188), clofazimine (21·8%, n = 41/188), cycloserine (12·8%, n = 24/188), delamanid (9·0%, n = 17/188), and pretomanid (0·53%, n = 1/188) (Figure S5B). The overall unavailability of phenotypic DST varied by individual drug, with pretomanid having the highest proportion of missing results (91·0%, n = 172/188), while moxifloxacin and linezolid had the lowest (1·6%, n = 3/188). In contrast, genotypic DST results were unavailable for more than 80% of patients across all reported drugs (linezolid, clofazimine, bedaquiline, delamanid, and pretomanid) (Table S4 in Supplementary Material). Phenotypic DST was less frequently performed in UMICs for cycloserine/terizidone, carbapenems, kanamycin, and ethionamide/prothionamide. Similarly, genotypic DST was less commonly performed in UMICs for linezolid, clofazimine, bedaquiline, pretomanid, and delamanid (Table S4 in Supplementary Material). Moreover, patients treated in UMICs were significantly older, had lower BMI, were less frequently smokers or intravenous drug users, and had more often been exposed to previous anti-tuberculous treatment (Tables S5 and S6 in Supplementary Material).

**Table 1:** Baseline socio-demographic characteristics and comorbidities of 188 extensively drug-resistant tuberculosis patients from 16 countries in the WHO European Region (2017–2023).

Characteristic	n/N (%)
Demographics	
Male	149/188 (79·3)
Age in years (at first visit), median (IQR)	42 (34, 52)
Age categories	
13–34 years	47/187 (25·1)
35–64 years	127/187 (67·9)
≥65 years	13/187 (7·0)
BMI (kg/m^2^), median (IQR)	21·0 (18·8, 23·0)
Social characteristics	
Active tobacco smoker	97/116 (83·6)
Active alcohol abuse	58/181 (32·0)
Homeless	22/166 (13·3)
Intravenous drug use	15/181 (8·3)
Comorbidities	
Chronic kidney disease	8/186 (4·3)
Diabetes mellitus type I or II	12/186 (6·5)
Active HBV infection	5/122 (4·1)
Active HCV infection	25/124 (20·2)
Living with HIV	27/185 (14·6)
Immunosuppression	8/184 (4·3)
Malignancy	4/186 (2·2)

Abbreviations: BMI, body mass index; HBV, hepatitis B virus; HCV, hepatitis C virus; HIV, human immunodeficiency virus 1.

**Table 2:** Baseline clinical characteristics of 188 extensively drug-resistant tuberculosis patients from 16 countries in the WHO Europe Region (2017–2023).

Characteristic	n/N (%)
TB disease characteristics	
TB localisation	
Extrapulmonary only	4/188 (2·1%)
Pulmonary only	170/188 (90·4%)
Pulmonary and extrapulmonary	14/188 (7·4%)
Bilateral lung involvement	115/160 (71·9%)
Any lung cavity	149/186 (80·1%)
Sputum smear positive	134/175 (76·6%)
Previous treatment	
Previous anti-TB treatment history	
None	37/182 (20·3%)
First-line drugs only	19/182 (10·4%)
Second-line drugs	126/182 (69·2%)
Current treatment	
Treatment duration in days, median (IQR)	382 (248, 567)
No. of drugs in the regimen, median (IQR)	6 (5, 8)
No. of likely effective drugs in the regimen, median (IQR)	3 (2, 4)

Abbreviations: TB, tuberculosis; IQR, interquartile range.

**Fig. 3:**
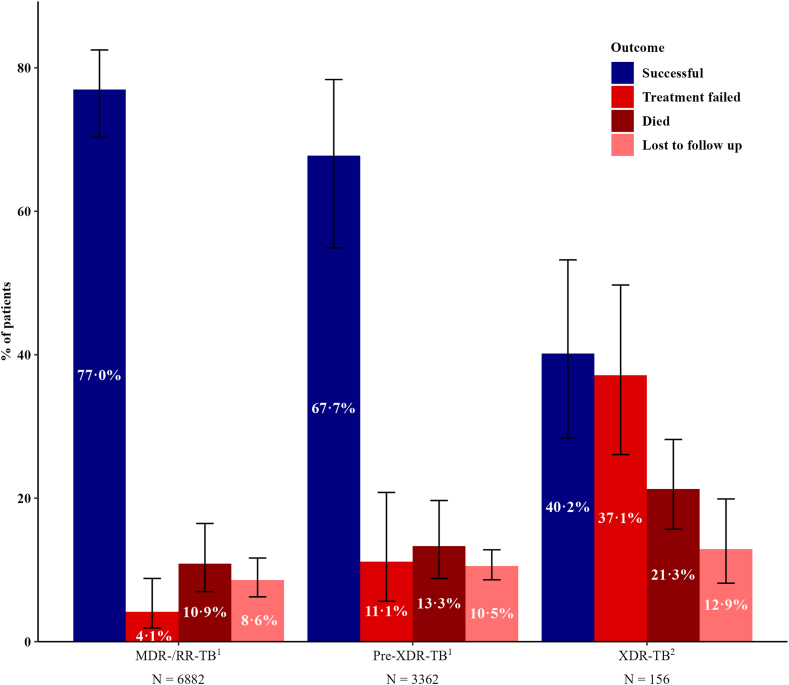
**Pooled percentages of treatment outcomes for patients with rifampicin-/multidrug-resistant, pre-extensively drug-resistant, and extensively drug-resistant tuberculosis in the WHO European Region (2017–2023), from a random-effects meta-analysis accounting for between-country variation.**^1^Based on aggregate data from 12 out of 18 participating centres. ^2^Based on patient-level data from the participating centres. Abbreviations: MDR-/RR-TB, multidrug/rifampicin-resistant tuberculosis; Pre-XDR-TB, pre-extensively drug-resistant tuberculosis; XDR-TB, extensively drug-resistant tuberculosis.

**Fig. 4:**
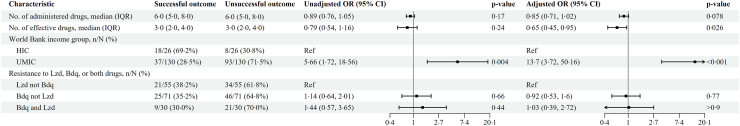
**Univariable and multivariable logistic regression analysis of risk factors for unsuccessful treatment outcome in 156 extensively drug-resistant tuberculosis patients from 16 countries in the WHO European Region (2017–2023). 95% confidence intervals were calculated using cluster-robust standard errors with clustering by country.** Odds ratios were adjusted for presence of lung cavities, previous treatment with second-line drugs, and sputum smear positivity. Abbreviations: OR, odds ratio; 95% CI, 95% confidence interval; HIC, high-income countries; UMIC, upper-middle-income countries; Lzd, linezolid; Bdq, bedaquiline.

Thirty-two patients (17·0%) were classified as ‘not evaluated’—including those still undergoing treatment—and were therefore excluded from outcome analyses. These excluded patients were significantly younger than those included in the analysis, but otherwise closely resembled the analysed population (Tables S7 and S8 in Supplementary Material). Among the 156 patients with extensively drug-resistant tuberculosis (XDR-TB) and available treatment outcomes, 55 experienced a successful outcome (Fig. 3). Crude percentages are available in Figure S6 in Supplementary Material. Based on a random-effects meta-analysis, the pooled percentage of successful outcomes was 40·2% (95% CI: 28·4–53·2) (Fig. 3). Among the 101 patients with unsuccessful treatment outcomes, treatment failure was most common (n = 57), corresponding to a pooled proportion of 37·1% (95% CI: 26·1–49·7). Death occurred in 30 patients, with a pooled proportion of 21·3% (95% CI: 15·7–28·2), and loss to follow-up was reported in 14 patients, corresponding to a pooled proportion of 12·9% (95% CI: 8·1–19·9). Based on aggregate data from 12 out of 18 participating centres, 5063 of 6880 patients with multidrug-/rifampicin-resistant tuberculosis experienced a successful treatment outcome, resulting in a pooled percentage of 77·0% (95% CI: 70·3–82·5). Among 3362 patients with pre-extensively drug-resistant tuberculosis, 2273 had a successful outcome, corresponding to a pooled estimate of 67·7% (95% CI: 58·1–76·1). Compared to patients with multidrug-/rifampicin-resistant- or pre-extensively drug-resistant tuberculosis, the pooled percentage of successful outcomes was significantly lower among patients with extensively drug-resistant tuberculosis (p < 0·0001), whereas the pooled percentages of treatment failure and death were significantly higher (p < 0·0001 and p = 0·008, respectively) (Figures S7–S10 in Supplementary Material). In the analysis of individual outcomes and different subgroups among patients with extensively drug-resistant tuberculosis, the pooled proportions of cure and treatment completion were significantly higher in HICs compared to UMICs (Fig. 4) (Figure S11 and Table S9 in Supplementary Material). Additionally, the pooled proportion of patients who died was significantly lower in patients who were treated with bedaquiline compared to those who were not. In terms of resistance to group A drugs, the pooled proportion of treatment failure was higher in patients with resistance to both linezolid and bedaquiline compared to those with resistance to either one of them, although the difference was not statistically significant (Fig. 4) (Figure S11 and Table S9 in Supplementary Material).

In total 124 patients had evidence of bedaquiline resistance. Of these, 74 still received bedaquiline, whereas 50 did not (Tables S10 and S11 in Supplementary Material). Baseline socio-demographic and clinical characteristics were generally comparable between the two groups. However, the patients with evidence of bedaquiline resistance who still received bedaquiline had a significantly higher proportion of adverse events (Table S11 in Supplementary Material). Among patients with microbiological evidence of bedaquiline resistance and an evaluated treatment outcome (n = 101), those who received bedaquiline (n = 62) had a pooled proportion of successful outcomes of 38·3% (95% CI: 20·1–60·5), which was not significantly different from the 43·2% (95% CI: 18·1–72·4) observed among those who did not receive bedaquiline (n = 39) (Figure S12 in Supplementary Material). In the multivariable logistic regression analysis, adjusting for lung cavitation, sputum smear positivity and previous treatment with second-line injectable drugs, the number of likely effective drugs in the regimen significantly reduced the odds of unsuccessful outcomes (aOR 0·65, 95% CI: 0·5, 0·9, p = 0·006), whereas being treated in an UMIC (aOR 13·7, 95% CI: 4·0, 56·6, p < 0·001) significantly increased the odds of unsuccessful outcomes compared to being treated in a HIC (Fig. 4).

**Fig. 5:**
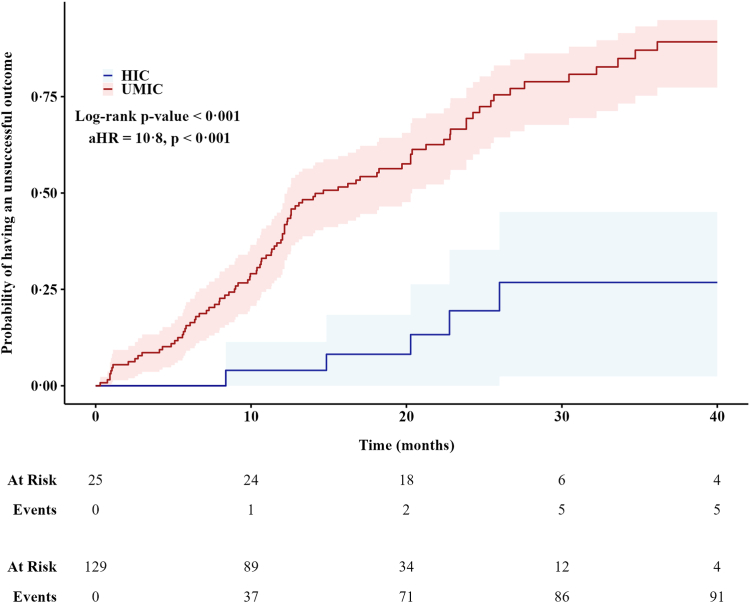
**Kaplan Meier curve of time to unsuccessful outcome for 154 extensively drug-resistant tuberculosis patients, stratified for World Bank income groups, in the WHO European Region (2017–2023).** Abbreviations: HIC, high-income country; UMIC, upper middle-income country.

The time to unsuccessful outcomes was significantly shorter in patients treated in UMICs compared with HICs (Log-rank p < 0·001) (Fig. 5). Similarly, time to death was shorter in UMICs compared with HICs, although the difference was not significant (Figure S13 in Supplementary Material).

In a sensitivity analysis, using an alternative definition of likely effective drugs yielded similar results for both the logistic and Cox regression analyses (Tables S12 and S13 in Supplementary Material).

## Discussion

Despite recent outstanding advancements in treatment for drug-resistant tuberculosis, our findings indicate that only 40% of patients affected by extensively drug-resistant tuberculosis in the WHO European region achieved successful outcomes, which is significantly lower than those for multidrug-resistant/rifampicin-resistant tuberculosis and pre-extensively drug-resistant tuberculosis.

These results are extremely concerning, as a pooled 40% success rate is comparable to the rate of spontaneous cure for tuberculosis from the pre-antibiotic era.^20^^,^^21^ Overall, this proportion of treatment success is lower than the pooled success rate of 44·8% in the WHO European region reported in a recent systematic review using the previous definition of extensively drug-resistant tuberculosis.^22^ However, it is also notably higher than the crude success rate of 31% reported in another recent observational study based on the new definition of extensively drug-resistant tuberculosis, which was conducted across five UMICs in Eastern Europe.^23^ The most likely explanation of this discrepancy is the more diverse representation of HICs in addition to UMICs in our study and the implementation of random effects to consider the resulting heterogeneity. Regardless, these success rates are remarkably far from WHO's End TB strategy targets, aiming for a 75% treatment success rate.^24^ The emergence of extensively drug-resistant tuberculosis poses an additional, major threat to meeting these targets.

Our study also underscores the critical need for optimised DST methods for early detection and adequate management of resistance to new and re-purposed anti-tuberculosis drugs, including fluoroquinolones, bedaquiline and linezolid. To date, rapid molecular tests are only available for fluoroquinolones and second-line injectables among second-line drugs. In parallel to the introduction of available tests, including targeted next generation sequencing, whole genome sequencing, and extended phenotypic DST capacity, new rapid diagnostic tools should be a research priority to allow for effective prevention and treatment of extensively drug-resistant tuberculosis.^25^ While the introduction of BPaLM and BPaL regimens has provided hope for improving multidrug-resistant/rifampicin-resistant tuberculosis and pre-extensively drug-resistant tuberculosis treatment outcomes, the real-world implementation of these regimens remains problematic.^11^ In our study, each additional likely effective drug significantly reduced the odds of unsuccessful outcomes, emphasising the importance of utilising drugs with proven susceptibility and ensuring their availability in all settings. Furthermore, we observed a significant disparity between UMICs and HICs in terms of access to drugs and DST, which may explain the finding of a higher likelihood of unfavourable outcome for patients treated in UMICs. This disparity highlights the urgent need for equitable access to tuberculosis treatments and advanced diagnostic tools across the WHO European Region. In addition, new compounds undergoing advanced stages of clinical development should be made available for the treatment of extensively drug-resistant tuberculosis patients with limited treatment alternatives. This could be done as part of compassionate use programs, building on experience from the early introduction of bedaquiline and delamanid.^11^^,^^26^, ^27^, ^28^ Comprehensive, global surveillance data on extensively drug-resistant tuberculosis outcomes are also necessary to monitor treatment progress and identify obstacles. The lack of specific guidelines and the complexity of interpreting phenotypic and genotypic DST results underscore the critical need to harmonise treatment approaches for extensively drug-resistant tuberculosis.

One of the major strengths of our study is the comprehensive assessment of the outcomes of extensively drug-resistant tuberculosis using the new definition across many countries of the WHO European region. This provides valuable insights into the effectiveness of current treatment regimens and highlights areas needing improvement. Our study demonstrates the importance of collaborative research through established clinical research networks, including countries and regions which are suffering from a long-term armed conflict.

Our study also has several limitations inherent to its retrospective design. Although many countries were included, coverage of the WHO European region was incomplete. Most participating centres were specialist or referral centres, which introduces a risk of selection bias and requires caution when generalising our results. However, given the complexity of extensively drug-resistant tuberculosis treatment, extensively drug-resistant tuberculosis patients are often managed in these settings, supporting the external validity of our findings within the European context. Although most variables were reported for all patients, missing values were common. Notably, 17% of extensively drug-resistant tuberculosis patients were not evaluated and were therefore excluded from the analyses. While this may also have introduced selection bias, the characteristics of the excluded patients were similar to those of the analysed population, suggesting that the risk was low.

The use of WHO-defined outcomes reduced the efforts required by clinicians to fill out the eCRF but also reduced the granularity of information, especially regarding treatment failure. This limited our ability to differentiate between microbiological failure, treatment modification due to adverse events, and regimen changes driven by drug resistance or unavailability. Furthermore, post-treatment follow-up other than treatment outcomes was not available in our study.

Additionally, while we compared treatment outcomes across multidrug-resistant/rifampicin-resistant, pre-extensively drug-resistant and extensively drug-resistant tuberculosis, individual-level data were only available for extensively drug-resistant tuberculosis patients. Therefore, the observed differences should be interpreted cautiously due to possible confounding by unmeasured patient-level factors. This is important considering that substantial heterogeneity was observed in the pooled proportions of successful outcomes for multidrug-resistant/rifampicin-resistant and pre-extensively drug-resistant tuberculosis. A notable proportion of patients had only genotypic DST results available, which may have led to misclassification of resistance status.

Finally, the low number of patients in the study only allowed including a limited number of covariates in the multivariable regression analyses. As a result, we were unable to adjust for some important potential confounders such as HIV status or undernutrition. Consequently, the observed associations between analysed covariates and outcomes may be influenced by unmeasured confounding.

### Conclusion

In conclusion, our findings reveal that treatment success rates for extensively drug-resistant tuberculosis in Europe remain discouragingly low, with a prognosis similar to tuberculosis in the pre-antibiotic era. These findings underscore the need for improved, rapid DST tools and effective, shorter treatment regimens for extensively drug-resistant tuberculosis. Meanwhile, widespread and parallel access to optimal DST and newer drugs is warranted globally. New anti-tuberculosis compounds should be made available through compassionate use and expanded access programs.^29^^,^^30^ Additionally, there is a pressing need to develop a framework for antibiotic stewardship in the context of drug-resistant tuberculosis to prevent the emergence and selection of further drug resistance, by ensuring for instance the parallel roll-out of DST capacities with marketing of novel medicines. In the long run, global collaboration and equitable resource distribution are critical to advancing the fight against drug-resistant tuberculosis and achieving the goals of the WHO End TB strategy.^31^

## Contributors

YK, OSP, CL, GG, and LG developed the concept of the study, developed the methodology and organised the data curation. GG and LG provided project supervision. All authors contributed to data acquisition. YK and OSP had access to all data and performed the data analysis and created the display items. YK, OSP, LG, and GG verified the data. YK, OSP, CL, GG, and LG wrote the initial draft. All authors participated in reviewing and editing. CL had the final responsibility for the decision to submit for publication.

## Data sharing statement

Data will be made available upon reasonable request, including for systematic reviews and meta-analyses. Requests should be directed to the corresponding author.

## Declaration of interests

LG is co-Principal Investigator of two MSF-sponsored Phase III randomised controlled clinical trials (endTB & endTB-Q) testing new shorter regimens for MDR/RR-TB. Both trials are mainly funded by Unitaid. LG is PI of a Phase III clinical trial (FAST-MDR) which is funded by the French National Hospital Program for Clinical Research (PHRC) and by a pro-bono donation by Viatris. CL is the clinical lead of the UNITE4TB consortium which conducts clinical tuberculosis trials in Phase IIa-c. CL received honoraria for speaking at symposia sponsored by Astra Zeneca, Gilead, GSK, Insmed, medUpdate, MedUpdateEurope, and Pfizer outside of the scope of this study. LRC received honoraria for speaking at symposia sponsored by Viatris and participated in an advisory board for Cepheid outside of the scope of this study. All other authors declare no competing interests.

## Figures and Tables

**Table:** 

Family name	First name	Affiliations
Alexandra	Aubry	1. Sorbonne Université, CNRS, INSERM, Centre d’Immunologie et des Maladies Infectieuses, CIMI, Paris 2. AP-HP.Sorbonne-Université, Centre National de Référence des Mycobactéries et de la Résistance des Mycobactéries aux Antituberculeux, Paris, France
François	Bénézit	Pontchaillou University Hospital, Infectious Disease and Intensive Care Unit, Rennes, France
Stephanie	Bjerrum	Department of Infectious Diseases, Rigshospitalet, University Hospital of Copenhagen, Denmark
Vivian	Bui Le	Centre of Excellence for Health, Immunity and Infections, Rigshospitalet, University of Copenhagen, Denmark
Dumitru	Chesov	1. Division of Clinical Infectious Diseases, Research Center Borstel, Borstel, Germany 2. German Center for Infection Research (DZIF), Partner Site Hamburg-Lübeck-Borstel-Riems, Borstel, Germany 3. Nicolae Testemitanu State University of Medicine and Pharmacy, Chisinau, Republic of Moldova 4. Chiril Draganiuc Pneumology Institute, Chisinau, Republic of Moldova
Daniela	Cirillo	Division of Immunology, Transplantation, and Infectious Diseases, IRCCS San Raffaele Scientific Institute, Milan, Italy. cirillo.daniela@hsr.it
Luigi Ruffo	Codecasa	Regional TB Reference Centre, Instituto Villa Marelli, ASST Grande Ospedale Metropolitano Niguarda, Milan, Italy
Roxana	Coriu	Marius Nasta Institute of Pneumology Bucharest, Bucharest, Romania
Edita	Davidavičienė	Department of Programs and State Tuberculosis Information System, Vilnius University Hospital Santaros Klinikos, Vilnius, Lithuania
Andrii	Dudnyk	1. Department of Tuberculosis, Clinical Immunology and Allergy, National Pirogov Memorial Medical University, Vinnytsia, Ukraine 2. Institut d'Investigació Germans Trias i Pujol (IGTP), Barcelona, Spain
Johannes	Eimer	Division of Infectious Diseases and Tropical Medicine, Department of Internal Medicine 4 Pneumology, Kepler University Hospital and Medical Faculty, Johannes Kepler University, Linz, Austria
Margaret	Fitzgibbon	Irish Mycobacteria Reference Laboratory, St James's Hospital, Dublin, Ireland
Giovanni	Fumagalli	1. STOP TB Italia, Milan, Italy 2. Respiratory Unit and TB Regional Reference Centre, Niguarda Hospital, University of Milan, Milan, Italy
Lorenzo	Guglielmetti	Department of Infectious, Tropical Diseases and Microbiology, IRCCS Sacro Cuore Don Calabria Hospital, Negrar di Valpolicella, Verona, Italy
Gunar	Günther	1. Department of Pulmonary Medicine, Allergology and Clinical Immunology, Inselspital, Bern University Hospital, University of Bern, Bern, Switzerland 2. Department of Medical Sciences, School of Medicine, University of Namibia, Windhoek, Namibia
Jerker	Jonsson	Department of Public Health Analysis and Data Management, Public Health Agency of Sweden, Stockholm, Sweden
Yousra	Kherabi	1. Infectious and Tropical Diseases Department, Bichat-Claude Bernard Hospital, Assistance Publique-Hôpitaux de Paris, Université Paris Cité, Paris, France 2. Université Paris Cité, Inserm, IAME, Paris, France
Nana	Kiria	National Center for Tuberculosis and Lung Diseases, Tbilisi, Georgia
Olha	Konstantynovska	1. V.N. Karazin Kharkiv National University, Department of Infectious Diseases and Clinical Immunology, Kharkiv, Ukraine 2. “Regional Phtisiopulmonological Center” of the Kharkiv Regional Council, Communal non-commercial enterprise, Kharkiv, Ukraine 3. Imperial College London, Department of Infectious Disease
Liga	Kuksa	Tuberculosis and Lung Disease clinic, Riga East Univesity hospital, Latvia
Christoph	Lange	1. Division of Clinical Infectious Diseases, Research Center Borstel, Borstel, Germany 2. German Center for Infection Research (DZIF), Partner Site Hamburg-Lübeck-Borstel-Riems, Borstel, Germany 3. Respiratory Medicine and International Health, University of Lübeck, Lübeck, Germany 4. Baylor College of Medicine and Texas Children Hospital, Global TB Program, Houston, TX, USA
Troels	Lillebaek	1. International Reference Laboratory of Mycobacteriology, Statens Serum Institut, Copenhagen, Denmark 2. Global Health Section, Department of Public Health, University of Copenhagen, Copenhagen, Denmark
Dhiba	Marigot-Outtandy	Sanatorium, Centre Hospitalier de Bligny, Briis-sous-Forges, France
Anne Marie	McLaughlin	Department of Respiratory Medicine, St James's Hospital, Dublin, Ireland
Ilaria	Motta	1. MRC Clinical Trials Unit at University College London, UK 2. Access Team, Médecins Sans Frontières, Geneva, Switzerland
Onya	Opota	Institute of Microbiology, Lausanne University Hospital and University of Lausanne, Lausanne, Switzerland
Traian-Constantin	Panciu	Marius Nasta Institute of Pneumology Bucharest, Bucharest, Romania
Daria	Podlekareva	International Reference Laboratory of mycobacteriology (IRLM), Statens Serum Institut, Denmark; Department of Respiratory- and Infectious Diseases Copenhagen University Hospital - Bispebjerg
Corentin	Poignon	1. Sorbonne-Université, INSERM, CNRS, Centre d’Immunologie et de Maladies Infectieuses, CIMI, Paris, France 2. Assistance Publique Hôpitaux de Paris, Groupe Hospitalier Universitaire Sorbonne Université, Hôpital Pitié-Salpêtrière, Centre National de Référence des Mycobactéries et de la Résistance des Mycobactéries aux Antituberculeux, Paris, France
Valérie	Pourcher	Assistance Publique Hôpitaux de Paris, Groupe Hospitalier Universitaire Sorbonne Université, Hôpital Pitié-Salpêtrière, Service des maladies infectieuses et tropicales
Matthieu	Revest	Infectious Diseases and Intensive Care Unit, Rennes Teaching Hospital, UMR 1230 Inserm, Rennes University, Rennes, France
Jérôme	Robert	1. Sorbonne Université, CNRS, INSERM, Centre d’Immunologie et des Maladies Infectieuses, CIMI, Paris 2. AP-HP.Sorbonne-Université, Centre National de Référence des Mycobactéries et de la Résistance des Mycobactéries aux Antituberculeux, Paris, France
Sten	Skogmar	1. Clinical Infection Medicine, Department of Translational Medicine, Lund University, Malmö 2. Department of Infectious Diseases, Skåne University Hospital, Malmö/Lund, Sweden
Ole Skouvig	Pedersen	1. Department of Respiratory Medicine and Allergy, Aarhus University Hospital, Aarhus, Denmark 2. Department of Clinical Medicine, Aarhus University, Aarhus, Denmark
Sirje	Sasi	Department of Microbiology and Molecular Diagnostics, Laboratory, North Estonia Medical Centre, Tallinn, Estonia
Dagmar	Schaub	1. Division of Clinical Infectious Diseases, Research Center Borstel, Borstel, Germany 2. German Center for Infection Research (DZIF), Partner Site Hamburg-Lübeck-Borstel-Riems, Borstel, Germany 3. Respiratory Medicine and International Health, University of Lübeck, Lübeck, Germany
Marcin	Skowroński	Wielkopolskie Centre of Pulmonology and Thoracic Surgery, Tuberculosis Department, Poznań, Poland
Varvara	Solodovnikova	TBnet, Bad Oldesloe, Germany
Tamar	Togonidze	National Center for Tuberculosis and Lung Diseases, Tbilisi, Georgia
Giuliana	Troia	Sanatorium, Centre Hospitalier de Bligny, Briis-sous-Forges, France
Simone	Tunesi	UOC Malattie infettive, Azienda Ospedaliera SS Antonio e Biagio e Cesare Arrigo, Alessandria, Italy
Laima	Vasiliauskaitè	1. Vilnius University, Faculty of Medicine, Institute of Biomedical Sciences, Department of Physiology, Biochemistry, Microbiology and Laboratory Medicine, Vilnius, Lithuania 2. Centre of Laboratory Medicine, Laboratory of Infectious Diseases and Tuberculosis, Vilnius University Hospital Santaros Klinikos, Vilnius, Lithuania
Anca	Vasiliu	1. Global TB Program, Department of Pediatrics, Baylor College of Medicie, Houston, United States 2. Clinical Infectious Diseases Department, Research Center Borstel, Germany 3. Clinical Tuberculosis Unit, German Center for Infection Research (DZIF), Hamburg-Lübeck-Borstel-Riems, Borstel D-23845, Germany
Nicolas	Veziris	1. Sorbonne Université, Centre d'Immunologie et des Maladies Infectieuses (Cimi-Paris), UMR 1135, Département de Bactériologie 2. Hôpital Saint-Antoine, Centre National de Référence des Mycobactéries, APHP. Sorbonne Université, Paris France
Piret	Viiklepp	Department of Registries, National Institute for Health Development, Talnn, Estonia
Lusine	Yeghiazaryan	National Center of Pulmonology, Abovyan, Republic of Armenia

## Appendix A Supplementary data

Supplement

Members of TBnet/ESCMYC Study Group

Supplementary Materials-Tables and Figures

eCRF
