# Supplementary material for: Treatment outcomes of extensively drug-resistant tuberculosis in Europe: a retrospective cohort study
Source: Lancet Reg Health Eur. 2025 Jul 15;56:101380. doi: 10.1016/j.lanepe.2025.101380 (PMC12281379; doi:10.1016/j.lanepe.2025.101380)
Supplement: Supplement [file mmc1.docx]

**Supplement**

**Kherabi Y et al.: Extensively drug-resistant tuberculosis in Europe. A TBnet/ESGMYC observational, retrospective cohort study**

**Ethic board approvals of the coordinating center and collaborative centers**

| **Country** | **Coordinating center** | **Local investigator** | **Ethics board** | **Ethics board approval** |
| --- | --- | --- | --- | --- |
| France | National Reference Center for Mycobacteria and Mycobacterial Resistance to Antituberculosis Agents, Paris, France | Yousra Kherabi | Comité d’éthique du sanatorium de Bligny, Bligny, France | Number  CR CRE 27/06/2023 |
| **Country** | **Collaborative centers** | **Local investigator** | **Ethics board** | **Ethics board approval** |
| Armenia | National Center of Pulmonology of the RA Ministry of Health CJSC | Lusine Yeghiazaryan | Ethical Board of the Yerevan State Medical University, Yerevan, Armenia | Not needed for retrospective anonymized data analysis. |
| Belarus | Republican Research and Practical Centre for Pulmonology and Tuberculosis | Varvara Solodovnikova | Ethics Committee of the Republican Research and Practical Centre for Pulmonology and Tuberculosis, Minsk, Belarus | Not needed for retrospective anonymized data analysis. |
| Denmark | Rigshospitalet, Department of Infectious Diseases | Stephanie Bjerrum | Capital Region of Denmark, Copenhagen, Denmark | Number  p-2024-15967 |
| Estonia | Tartu University Hospital; North Estonia Medical Centre | Sirje Sasi (PI),  Kaarel Kisant (represents Tartu) | Tervise Arengu Instituudi inimuuringute eetikakomitee, Tartu, Estonia | Number 1320 |
| Georgia | National Center for Tuberculosis and Lung Diseases | Nana Kiria | Ethical Board of National Center for Tuberculosis and Lung Diseases, Tbilisi, Georgia | Number 554/05 |
| Germany | Research Center Borstel, Leibniz Lung Center | Christoph Lange | Ethikkommission der Universität zu Lübeck, Lübeck, Germany | Number 2024/239 |
| Ireland | Ireland- St James's Hospital | Margaret Fitzgibbon | Ethical Board of Trinity College Dublin, Dublin, Ireland | Number: 3509 |
| Italy | Villa Marelli Regional TB Ref Centre, Niguarda Hospital | Luigi Ruffo Codecasa | Lombardy EC3 Ethical Board | Number 5749_SA_06.12.2024_N |
| Latvia | Riga East University hospital, TB and Lung disease clinic | Liga Kuksa | Riga East University hospital Ethics committee, Riga, Latvia | Not needed for retrospective anonymized data analysis |
| Lithuania | Infectious Diseases and Tuberculosis Laboratory (Lithuanian National Reference Laboratory for TB), Centre of Laboratory medicine, Vilnius University Hospital Santaros Klinikos | Laima Vasiliauskaitė | Ethical Board of Vilnius University, Vilnius, Lithuania | Number  2024/6-1596-1053 |
| Moldova | Department of Pneumology & Allergology, State University of Medicine and Pharmacy "Nicolae Testemitanu" | Dumitru Chesov | Ethical Board of Nicolae Testemitanu USMPh, Chisinau, R. Moldova | Number 3/ 10.07.2024 |
| Poland | Wielkopolska Center of Pulmonology and Thoracic Surgery | Marcin Skowroński | Ethical Board of University of Medical Sciences, Poznan, Poland | Not needed for retrospective anonymized data analysis. |
| Romania | Marius Nasta Institute of Pneumophtiziology | Traian Constantin Panciu | Local Ethical Board of Marius Nasta Institute, Bucharest, Romania | Number 1824/18.01.2024 |
| Ukraine | Regional Phthisiopulmonological Center, Vinnytsia | Andrii Dudnyk | The Bioethics Committee of the National Pirogov Memorial Medical University, Vinnytsia, Ukraine | Not needed for retrospective anonymized data analysis. |
| Ukraine | Regional Tuberculosis Dispensary #1, Kharkiv | Olha Konstantynovska | V.N.Karazin Kharkiv National University, Department of Infectious Diseases and Clinical Immunology, Kharkiv, Ukraine | Not needed for retrospective anonymized data analysis |
