# Supplementary material for: Treatment outcomes of extensively drug-resistant tuberculosis in Europe: a retrospective cohort study
Source: Lancet Reg Health Eur. 2025 Jul 15;56:101380. doi: 10.1016/j.lanepe.2025.101380 (PMC12281379; doi:10.1016/j.lanepe.2025.101380)
Supplement: Members of TBnet/ESCMYC Study Group [file mmc2.docx]

**Two column table of additional members of the TBnet/ESCMYC XDR-TB study group**

| **Alexandra** | **Aubry** |
| --- | --- |
| **Stephanie** | **Bjerrum** |
| **Vivian** | **Bui Le** |
| **Daniela** | **Cirillo** |
| **Roxana** | **Coriu** |
| **Edita** | **Davidavičienė** |
| **Johannes** | **Eimer** |
| **Margaret** | **Fitzgibbon** |
| **Giovanni** | **Fumagalli** |
| **Jerker** | **Jonsson** |
| **Liga** | **Kuska,** |
| **Troels** | **Lillebaek** |
| **Anne Marie** | **McLaughlin** |
| **Ilaria** | **Motta** |
| **Onya** | **Opota** |
| **Daria** | **Podlekareva** |
| **Valérie** | **Pourcher** |
| **Mathieu** | **Revest** |
| **Jérôme** | **Robert** |
| **Sten** | **Skogmar** |
| **Marcin** | **Skowroński** |
| **Tamar** | **Togonidze** |
| **Giuliana** | **Troia** |
| **Simone** | **Tunesi** |
| **Anca** | **Vasiliu** |
| **Nicolas** | **Veziris** |
| **Piret** | **Viiklepp** |
