# Supplementary material for: Treatment outcomes of extensively drug-resistant tuberculosis in Europe: a retrospective cohort study
Source: Lancet Reg Health Eur. 2025 Jul 15;56:101380. doi: 10.1016/j.lanepe.2025.101380 (PMC12281379; doi:10.1016/j.lanepe.2025.101380)
Supplement: Supplementary Materials-Tables and Figures [file mmc3.docx]

**SUPPLEMENTARY MATERIAL**

**Table S1.** STROBE Checklist for Cohort Studies.

STROBE Statement—Checklist of items that should be included in reports of ***cohort studies***

|  | Item No | Recommendation | Page no. |
| --- | --- | --- | --- |
| **Title and abstract** | 1 | (*a*) Indicate the study’s design with a commonly used term in the title or the abstract | 3 |
|  |  | (*b*) Provide in the abstract an informative and balanced summary of what was done and what was found | 3 |
| Introduction | | |  |
| Background/rationale | 2 | Explain the scientific background and rationale for the investigation being reported | 7-8 |
| Objectives | 3 | State specific objectives, including any prespecified hypotheses | 8 |
| Methods | | |  |
| Study design | 4 | Present key elements of study design early in the paper | 8 |
| Setting | 5 | Describe the setting, locations, and relevant dates, including periods of recruitment, exposure, follow-up, and data collection | 8-9 + Supplementary Figure 1 |
| Participants | 6 | (*a*) Give the eligibility criteria, and the sources and methods of selection of participants. Describe methods of follow-up | 8-9 |
|  |  | (*b*) For matched studies, give matching criteria and number of exposed and unexposed |  |
| Variables | 7 | Clearly define all outcomes, exposures, predictors, potential confounders, and effect modifiers. Give diagnostic criteria, if applicable | 9-10 |
| Data sources/ measurement | 8* | For each variable of interest, give sources of data and details of methods of assessment (measurement). Describe comparability of assessment methods if there is more than one group | 8 |
| Bias | 9 | Describe any efforts to address potential sources of bias | 9-10 |
| Study size | 10 | Explain how the study size was arrived at | NA |
| Quantitative variables | 11 | Explain how quantitative variables were handled in the analyses. If applicable, describe which groupings were chosen and why | 9 |
| Statistical methods | 12 | (*a*) Describe all statistical methods, including those used to control for confounding | 9-10 |
|  |  | (*b*) Describe any methods used to examine subgroups and interactions | 9-10 |
|  |  | (*c*) Explain how missing data were addressed | 9-10 |
|  |  | (*d*) If applicable, explain how loss to follow-up was addressed | 9-10 |
|  |  | (*e*) Describe any sensitivity analyses | 10 |
| Results | | |  |
| Participants | 13* | (a) Report numbers of individuals at each stage of study—eg numbers potentially eligible, examined for eligibility, confirmed eligible, included in the study, completing follow-up, and analysed | 10 and Figure 1 |
|  |  | (b) Give reasons for non-participation at each stage | Figure 1 |
|  |  | (c) Consider use of a flow diagram | Figure 1 |
| Descriptive data | 14* | (a) Give characteristics of study participants (eg demographic, clinical, social) and information on exposures and potential confounders | Table 1 and 2 |
|  |  | (b) Indicate number of participants with missing data for each variable of interest | Table 1 and 2 |
|  |  | (c) Summarise follow-up time (eg, average and total amount) | NA |
| Outcome data | 15* | Report numbers of outcome events or summary measures over time | 11-12 |
| Main results | 16 | (*a*) Give unadjusted estimates and, if applicable, confounder-adjusted estimates and their precision (eg, 95% confidence interval). Make clear which confounders were adjusted for and why they were included | Figure 6 |
|  |  | (*b*) Report category boundaries when continuous variables were categorized | NA |
|  |  | (*c*) If relevant, consider translating estimates of relative risk into absolute risk for a meaningful time period | NA |
| Other analyses | 17 | Report other analyses done—eg analyses of subgroups and interactions, and sensitivity analyses | 12-13 |
| Discussion | | |  |
| Key results | 18 | Summarise key results with reference to study objectives | 13 |
| Limitations | 19 | Discuss limitations of the study, taking into account sources of potential bias or imprecision. Discuss both direction and magnitude of any potential bias | 14 |
| Interpretation | 20 | Give a cautious overall interpretation of results considering objectives, limitations, multiplicity of analyses, results from similar studies, and other relevant evidence | 14-15 |
| Generalisability | 21 | Discuss the generalisability (external validity) of the study results | 14 |
| Other information | | |  |
| Funding | 22 | Give the source of funding and the role of the funders for the present study and, if applicable, for the original study on which the present article is based | 26 |

*Give information separately for exposed and unexposed groups.

**Note:** An Explanation and Elaboration article discusses each checklist item and gives methodological background and published examples of transparent reporting. The STROBE checklist is best used in conjunction with this article (freely available on the Web sites of PLoS Medicine at http://www.plosmedicine.org/, Annals of Internal Medicine at http://www.annals.org/, and Epidemiology at http://www.epidem.com/). Information on the STROBE Initiative is available at http://www.strobe-statement.org.

**Table S2**. Treatment outcome definitions.

| **Outcome** | **Definition** |
| --- | --- |
| Cured | Treatment completed as recommended by the national policy without evidence of failure AND three or more consecutive cultures taken at least 30 days apart are negative after the intensive phase. |
| Treatment completed | Treatment completed as recommended by the national policy without evidence of failure BUT no record that three or more consecutive cultures taken at least 30 days apart are negative after the intensive phase. |
| Treatment failed | Treatment terminated or need for permanent regimen change of at least two anti-TB drugs because of:  − lack of conversion by the end of the intensive phase, or  − bacteriological reversion in the continuation phase after conversion to negative, or  − evidence of additional acquired resistance to fluoroquinolones or second-line injectable drugs, or  − adverse drug reactions (ADRs) |
| Died | A patient who dies for any reason during the course of treatment. |
| Lost to follow-up | A patient whose treatment was interrupted for 2 consecutive months or more. |
| Not evaluated | A patient for whom no treatment outcome is assigned. (This includes cases “transferred out” to another treatment unit and whose treatment outcome is unknown) |
| Successful treatment outcome | The sum of *cured* and *treatment completed* |
| Unsuccessul treatment outcome | The sum of *treatment failed*, *died*, and *lost to follow-up* |

Modified from Table A.2.2. in *Definitions and reporting framework for tuberculosis – 2013 revision (updated December 2014 and January 2020)****.***

**Table S3**. Drugs included in treatment regimens, by country income category.

| **Characteristic** | **HIC**  N = 40 | **UMIC**  N = 148 | **p-value**^1^ |
| --- | --- | --- | --- |
| Median no. of drugs in current regimen (IQR) | 7 (6, 8) | 6 (5, 7) | 0.007 |
| Lfx | 3/40 (7.5%) | 31/148 (21%) | 0.050 |
| Mfx | 18/40 (45%) | 24/148 (16%) | <0.001 |
| Bdq | 29/40 (73%) | 93/148 (63%) | 0.3 |
| Lzd | 32/40 (80%) | 119/148 (80%) | >0.9 |
| Pa | 6/40 (15%) | 4/148 (2.7%) | 0.007 |
| Cfz | 33/40 (83%) | 109/148 (74%) | 0.2 |
| Cs/Tzd | 31/40 (78%) | 116/148 (78%) | >0.9 |
| E | 9/40 (23%) | 28/148 (19%) | 0.6 |
| Dlm | 33/40 (83%) | 104/148 (70%) | 0.12 |
| Z | 11/40 (28%) | 66/148 (45%) | 0.051 |
| Carb + Amx/Clv | 23/40 (58%) | 73/148 (49%) | 0.4 |
| Amk | 25/40 (63%) | 32/148 (22%) | <0.001 |
| Km | 0/40 (0%) | 7/148 (4.7%) | 0.3 |
| Cm | 3/40 (7.5%) | 24/148 (16%) | 0.2 |
| Eto/Pto | 10/40 (25%) | 40/148 (27%) | 0.8 |
| PAS | 14/40 (35%) | 44/148 (30%) | 0.5 |
| Other drugs | 5/40 (13%) | 23/148 (16%) | 0.6 |
| ^1^Wilcoxon rank sum test; Pearson's Chi-squared test; Fisher's exact test  Abbreviations: HIC, high-income country ; UMIC, upper-middle-income country; Lfx, levofloxacin; Mfx, moxifloxacin; Bdq, bedaquiline; Lzd, linezolid; Cfz, clofazimine; Cs/Tzd, cycloserine/terizidone; E, ethambutol; Dlm, delamanid; Pa, pretomanid; Z, pyrazinamide; Carb + Amx/Clav, carbapenem + amoxicillin/clavulanic acid; Amk, amikacin; Cm, capreomycin; Km, kanamycin; Eto/Pto, ethionamide/prothionamide; PAS, P-aminosaliscylic acid. | | | |

**Table S4.** Availability of drug susceptibility testing results, by country income category.

| **Characteristic** | **Overall**  N = 188 | **HIC**  N = 40 | **UMIC**  N = 148 | **p-value**^1^ |
| --- | --- | --- | --- | --- |
| Median number of drugs where DST was not performed |  | 5.00 (3.00, 6.50) | 5.00 (4.00, 7.00) | 0.078 |
| *Phenotypic DST not performed, n/N (%)* |  |  |  |  |
| Lfx | 9/188 (4.8%) | 8/40 (20%) | 1/148 (0.7%) | <0.001 |
| Mfx | 3/188 (1.6%) | 0/40 (0%) | 3/148 (2.0%) | >0.9 |
| Bdq | 14/188 (7.4%) | 5/40 (13%) | 9/148 (6.1%) | 0.2 |
| Lzd | 3/188 (1.6%) | 1/40 (2.5%) | 2/148 (1.4%) | 0.5 |
| Pa | 172/188 (91%) | 36/40 (90%) | 136/148 (92%) | 0.8 |
| Cfz | 65/188 (35%) | 22/40 (55%) | 43/148 (29%) | 0.002 |
| Cs/Tzd | 90/188 (48%) | 12/40 (30%) | 78/148 (53%) | 0.011 |
| E | 7/188 (3.7%) | 2/40 (5.0%) | 5/148 (3.4%) | 0.6 |
| Dlm | 76/188 (40%) | 12/40 (30%) | 64/148 (43%) | 0.13 |
| Z | 34/188 (18%) | 8/40 (20%) | 26/148 (18%) | 0.7 |
| Carb + Amx/Clv | 186/188 (99%) | 38/40 (95%) | 148/148 (100%) | 0.044 |
| Amk | 16/188 (8.5%) | 2/40 (5.0%) | 14/148 (9.5%) | 0.5 |
| Cm | 60/188 (32%) | 12/40 (30%) | 48/148 (32%) | 0.8 |
| Km | 124/188 (66%) | 17/40 (43%) | 107/148 (72%) | <0.001 |
| Eto/Pto | 48/188 (26%) | 3/40 (7.5%) | 45/148 (30%) | 0.003 |
| PAS | 96/188 (51%) | 21/40 (53%) | 75/148 (51%) | 0.8 |
| *Genotypic DST not performed, n/N (%)* |  |  |  |  |
| Lzd | 156/188 (83%) | 8/40 (20%) | 148/148 (100%) | <0.001 |
| Cfz | 158/188 (84%) | 10/40 (25%) | 148/148 (100%) | <0.001 |
| Bdq | 156/188 (83%) | 8/40 (20%) | 148/148 (100%) | <0.001 |
| Dlm | 174/188 (93%) | 26/40 (65%) | 148/148 (100%) | <0.001 |
| Pa | 185/188 (98%) | 37/40 (93%) | 148/148 (100%) | 0.009 |
| ^1^Wilcoxon rank sum test; Fisher's exact test; Pearson's Chi-squared test  Abbreviations: HIC, high-income country; UMIC, upper-middle-income country; Lfx, levofloxacin; Mfx, moxifloxacin; Bdq, bedaquiline; Lzd, linezolid; Cfz, clofazimine; Cs/Tzd, cycloserine/terizidone; E, ethambutol; Dlm, delamanid; Pa, pretomanid; Z, pyrazinamide; Carb + Amx/Clav, carbapenem + amoxicillin/clavulanic acid; Amk, amikacin; Cm, capreomycin; Km, kanamycin; Eto/Pto, ethionamide/prothionamide; PAS, P-aminosaliscylic acid. | | | | |

**Table S5.**  Baseline socio-demographic characteristics and comorbidities of patients with extensively drug-resistant tuberculosis, by country income category.

| **Characteristic** | **HIC**  n/N (%) | **UMIC**  n/N (%) | **p-value**^1^ |
| --- | --- | --- | --- |
| Demographics |  |  |  |
| Male | 31/40 (78) | 118/148 (80) | 0·8 |
| Age in years (at first visit), median (IQR) | 38 (32, 49) | 43 (36, 55) | 0·025 |
| Age categories |  |  | 0·020 |
| 13-34 years | 16/40 (40) | 27/147 (18) |  |
| 35-64 years | 22/40 (55) | 108/147 (73) |  |
| ≥ 65 years | 2/40 (5·0) | 12/147 (8·2) |  |
| Social characteristics |  |  |  |
| BMI (kg/m^2^), median (IQR) | 22 (20, 55) | 20 (19, 22) | 0·003 |
| Active tobacco smoker | 11/27 (41) | 11/139 (7·9) | <0·001 |
| Active alcohol abuse | 27/35 (77) | 70/81 (86) | 0·2 |
| Homeless | 9/34 (26) | 49/147 (33) | 0·4 |
| Intravenous drug use | 6/35 (17) | 9/146 (6·2) | 0·045 |
| Comorbidities |  |  |  |
| Chronic kidney disease | 2/39 (5·1) | 6/147 (4·1) | 0·7 |
| Diabetes mellitus type I or II | 1/39 (2·6) | 11/147 (7·5) | 0·5 |
| Active HBV infection | 1/38 (2·6) | 4/84 (4·8) | >0·9 |
| Active HCV infection | 8/37 (22) | 17/87 (20) | 0·8 |
| Living with HIV | 6/39 (15) | 21/146 (14) | 0·9 |
| Immunosuppression | 2/38 (5·3) | 6/146 (4·1) | 0·7 |
| Malignancy | 2/39 (5·1) | 2/147 (1·4) | 0·2 |
| ^1^Pearson's Chi-squared test; Wilcoxon rank sum test; Fisher's exact test.  Abbreviations: HIC, high income country; UMIC, upper-middle income country; BMI, body mass index; HBV, hepatitis B virus; HCV, hepatitis C virus; HIV, human immunodeficiency virus 1. | | | |

**Table S6.** Baseline clinical characteristics, stratified by country income category.

| **Characteristic** | **HIC**  n/N (%) | **UMIC**  n/N (%) | **p-value**^1^ |
| --- | --- | --- | --- |
| TB disease characteristics |  |  |  |
| TB localization |  |  | >0·9 |
| Extrapulmonary only | 1/40 (2·5%) | 3/148 (2·0%) |  |
| Pulmonary only | 36/40 (90·0%) | 134/148 (90·5%) |  |
| Pulmonary and extrapulmonary | 3/40 (7·5%) | 11/148 (7·4%) |  |
| Bilateral lung involvement | 23/27 (85·2%) | 92/133 (69·2%) | 0·092 |
| Any lung cavity | 32/38 (84·2%) | 117/148 (79·1%) | 0·5 |
| Sputum smear positive | 27/30 (90·0%) | 107/145 (73·8%) | 0·056 |
| Previous treatment |  |  |  |
| Previous anti-TB treatment history |  |  | 0·016 |
| None | 11/35 (31·4%) | 26/147 (17·7%) |  |
| First-line drugs only | 0/35 (0·0%) | 19/147 (12·9%) |  |
| Second-line drugs | 24/35 (68·6%) | 102/147 (69·4%) |  |
| Current treatment |  |  |  |
| Treatment duration in days, median (IQR) | 617·0 (452·0, 734·0) | 365·0 (233·0, 539·0) | <0·001 |
| No. of drugs in the regimen, median (IQR) | 7·0 (6·0, 8·0) | 6·0 (5·0, 7·0) | 0·007 |
| No. of likely effective drugs in the regimen, median (IQR) | 3·0 (1·5, 4·0) | 3·0 (2·0, 4·0) | >0·9 |
| ^1^Fisher's exact test; Pearson's Chi-squared test; Wilcoxon rank sum test.  Abbreviations: HIC, high income country; UMIC, upper-middle income country; TB, tuberculosis; IQR, interquartile range. | | | |

**Table S7.** Baseline socio-demographic characteristics and comorbidities, stratified by inclusion in the final analysis.

| **Characteristic** | **Not evaluated**  N = 32 | **Included in analyses** N = 156 | **p-value**^1^ |
| --- | --- | --- | --- |
| Male | 27/32 (84) | 122/156 (78) | 0.4 |
| Age in years (at first visit), median (IQR) | 37 (32, 45) | 43 (36, 56) | 0.009 |
| Age categories |  |  | 0.074 |
| 13-34 years | 11/32 (34) | 32/155 (21) |  |
| 35-64 years | 21/32 (66) | 109/155 (70) |  |
| ≥ 65 years | 0/32 (0) | 14/155 (9.0) |  |
| Body mass index (kg/m^2^), median (IQR) | 20 (18, 22) | 21 (19, 23) | 0.3 |
| Homelessness | 6/25 (24) | 16/141 (11) | 0.11 |
| Active tobacco smoker | 20/26 (77) | 77/90 (86) | 0.4 |
| Active alcohol abuse | 12/29 (41) | 46/152 (30) | 0.2 |
| Intravenous drug abuse | 4/29 (14) | 11/152 (7.2) | 0.3 |
| HIV | 7/31 (23) | 20/154 (13) | 0.2 |
| HBV | 0/28 (0) | 5/94 (5.3) | 0.6 |
| HCV | 3/27 (11) | 22/97 (23) | 0.2 |
| Immunosuppression | 3/31 (9.7) | 5/153 (3.3) | 0.13 |
| Diabetes mellitus type I or II | 1/31 (3.2) | 11/155 (7.1) | 0.7 |
| Malignancy | 0/31 (0) | 4/155 (2.6) | >0.9 |
| Chronic kidney disease | 1/31 (3.2) | 7/155 (4.5) | >0.9 |
| Hypoalbuminemia | 2/16 (13) | 20/73 (27) | 0.3 |
| ^1^Pearson's Chi-squared test; Wilcoxon rank sum test; Fisher's exact test.  Abbreviations: HIC, high income country; UMIC, upper-middle income country; BMI, body mass index; HBV, hepatitis B virus; HCV, hepatitis C virus; HIV, human immunodeficiency virus 1. | | | |

**Table S8.** Baseline clinical characteristics, stratified by inclusion in the final analysis.

| **Characteristic** | **Not evaluated**  N = 32 | **Included in analyses**  N = 156 | **p-value**^1^ |
| --- | --- | --- | --- |
| TB localization |  |  | 0.6 |
| Extrapulmonary only | 1/32 (3.1%) | 3/156 (1.9%) |  |
| Pulmonary | 28/32 (87.5%) | 142/156 (91.0%) |  |
| Pulmonary and extrapulmonary | 3/32 (9.4%) | 11/156 (7.1%) |  |
| Bilateral lung involvement | 19/25 (76.0%) | 96/135 (71.1%) | 0.6 |
| Any lung cavity | 24/30 (80.0%) | 125/156 (80.1%) | >0.9 |
| Smear positive | 21/27 (77.8%) | 113/148 (76.4%) | 0.9 |
| Previous anti-TB treatment history |  |  | 0.053 |
| None | 11/32 (34.4%) | 26/150 (17.3%) |  |
| First-line drugs | 1/32 (3.1%) | 18/150 (12.0%) |  |
| Second-line drugs | 20/32 (62.5%) | 106/150 (70.7%) |  |
| Yes, but regimen is unkown | 0/32 (0.0%) | 0/150 (0.0%) |  |
| Treatment duration in days, median (IQR) | 249.0 (130.0, 365.0) | 391.0 (255.0, 567.0) | 0.081 |
| No. of drugs administered, median (IQR) | 6.0 (5.0, 7.0) | 6.0 (5.0, 8.0) | 0.6 |
| No. of effective drugs, median (IQR) | 3.0 (2.0, 4.0) | 3.0 (2.0, 4.0) | 0.6 |
| Any adverse events | 11/32 (34.4%) | 35/156 (22.4%) | 0.2 |
| ^1^Fisher's exact test; Pearson's Chi-squared test; Wilcoxon rank sum test  Abbreviations: HIC, high income country; UMIC, upper-middle income country; TB, tuberculosis; IQR, interquartile range. | | | |

**Table S9.** Pooled percentages and 95% confidence intervals with country-specific random effects for each successful and unsuccessful outcome for different subgroups of patients (n=156), corresponding to the percentages in Figure S10.

| **Group** | **Cured,** n/N (95% | **Treatment completed** | **Died** | **Treatment failed** | **Lost to follow up** |
| --- | --- | --- | --- | --- | --- |
| World Bank income group |  |  |  |  |  |
| HIC | 13/26 (50·0%, 33·9–66·1) | 5/26 (27·0%, 14·3–45·1) | 5/26 (25·7%, 9·7–52·7) | 2/26 (21·5%, 9·5–41·5) | 1/26 (14·1%, 7·9–23·8) |
| UMIC | 30/130 (24·1%, 16·1–34·5)* | 7/130 (7·4%, 1·8–26·0)* | 25/130 (20·5%, 14·4–28·3) | 55/130 (41·2%, 24·3–60·5) | 13/130 (13·6%, 5·2–30·9) |
| No. of effective drugs |  |  |  |  |  |
| ≤ 1 | 4/29 (23·7%, 11·8–41·9) | 2/29 (17·9%, 5·6–44·3) | 8/29 (28·0%, 13·3–49·7) | 13/29 (47·8%, 20·8–76·1) | 2/29 (16·3%, 8·1–30·0) |
| 2 | 12/39 (34·4%, 17·8–56·0) | 2/39 (15·6%, 8·7–25·6) | 7/39 (23·8%, 16·4–33·2) | 16/39 (44·3%, 32·0–57·4) | 2/39 (15·8%, 6·8–32·7) |
| 3 | 12/37 (37·8%, 18·2–62·4) | 3/37 (18·6%, 10·2–31·3) | 7/37 (28·3%, 15·2–46·5) | 11/37 (32·4%, 22·4–44·4) | 4/37 (16·3%, 9·1–27·4) |
| ≥ 4 | 15/51 (33·3%, 21·5–47·7) | 5/51 (23·5%, 10·0–46·0) | 8/51 (19·5%, 13·0–28·4) | 17/51 (36·9%, 21·9–54·9) | 6/51 (16·1%, 10·6–23·6) |
| Bdq included in regimen |  |  |  |  |  |
| No | 15/52 (34·9%, 14·6–62·5) | 1/52 (12·3%, 3·9–32·6) | 13/52 (29·4%, 19·3–42·0) | 19/52 (38·3%, 25·4–53·0) | 4/52 (15·8%, 7·0–31·7) |
| Yes | 28/104 (30·1%, 19·6–43·2) | 11/104 (19·0%, 9·9–33·3) | 17/104 (17·5%, 11·8–25·2)* | 38/104 (34·0%, 19·9–51·6) | 10/104 (13·6%, 9·2–19·7) |
| Bdq and/or Lzd resistance |  |  |  |  |  |
| Bdq and Lzd | 7/30 (29·4%, 17·3–45·4) | 2/30 (20·9%, 9·2–40·7) | 4/30 (22·4%, 14·6–29·6) | 16/30 (49·7%, 29·0–70·0) | 1/30 (17·5%, 8·8–31·6) |
| Bdq not Lzd | 21/71 (31·5%, 18·9–46·1) | 4/71 (13·1%, 5·3–29·9) | 16/71 (24·7%, 15·7–36·6) | 21/71 (35·1%, 26·3–48·6) | 9/71 (16·4%, 10·2–25·3) |
| Lzd not Bdq | 15/55 (34·0%, 15·5–59·1) | 6/55 (20·3%, 11·6–33·1) | 10/55 (20·8%, 14·0–29·8) | 20/55 (38·2%, 28·3–49·3) | 4/55 (13·0%, 8·0–20·6) |

Abbreviations: 95% CI, 95% confidence interval; HIC, high-income countries; UMIC, upper-middle-income countries; Lzd, linezolid; Bdq, bedaquiline
*Chi-squared P-value for subgroup differences < 0.05

**Table S10.** Comparison of socio-demographic factors, comorbidities, and behavioral characteristics among patients with evidence of bedaquiline resistance stratified by treatment with bedaquline (n = 124).

| **Characteristic** | **Did not receive BDQ**  N = 50 | **Received BDQ**  N = 74 | **p-value**^1^ |
| --- | --- | --- | --- |
| Demographics, n/N (%) |  |  |  |
| Male | 36/50 (72) | 62/74 (84) | 0·11 |
| Age in years (at first visit), median (IQR) | 42 (32, 53) | 43 (34, 53) | 0·8 |
| Body mass index (kg/m^2^), median (IQR) | 21 (19, 23) | 21 (19, 24) | 0·7 |
| Country income group |  |  | 0·2 |
| HIC | 10/50 (20) | 22/74 (30) |  |
| UMIC | 40/50 (80) | 52/74 (70) |  |
| Social characteristics, n/N (%) |  |  |  |
| Homelessness | 3/45 (6·7) | 12/65 (18) | 0·076 |
| Active tobacco smoker | 18/25 (72) | 40/46 (87) | 0·2 |
| Active alcohol abuse | 9/48 (19) | 23/70 (33) | 0·090 |
| Intravenous drug abuse | 2/48 (4·2) | 3/70 (4·3) | >0·9 |
| Comorbidities n/N (%) |  |  |  |
| HIV | 6/49 (12) | 10/72 (14) | 0·8 |
| HBV | 1/28 (3·6) | 1/48 (2·1) | >0·9 |
| HCV | 8/30 (27) | 5/47 (11) | 0·067 |
| Immunosuppression | 0/49 (0) | 1/73 (1·4) | >0·9 |
| Diabetes mellitus type I or II | 4/49 (8·2) | 2/73 (2·7) | 0·2 |
| Malignancy | 1/49 (2·0) | 2/74 (2·7) | >0·9 |
| Chronic kidney disease | 1/49 (2·0) | 3/74 (4·1) | >0·9 |
| ^1^Pearson's Chi-squared test; Wilcoxon rank sum test; Fisher's exact test.  Abbreviations: BDQ, bedaquiline; IQR, interquartile range; HIC, high income country; UMIC, upper-middle income country; BMI, body mass index; HBV, hepatitis B virus; HCV, hepatitis C virus; HIV, human immunodeficiency virus 1. | | | |

**Table S11.** Comparison of treatment outcomes and clinical characteristics among patients with evidence of bedaquiline resistance stratified by treatment with bedaquline (n = 124).

| **Characteristic** | **Did not receive BDQ**  N = 50 | **Received BDQ**  N = 74 | **p-value**^1^ |
| --- | --- | --- | --- |
| Treatment outcome (n=101)^a^ |  |  | >0·9 |
| Successful | 13/39 (33·3) | 21/62 (33·9) |  |
| Unsuccessful | 26/39 (66·7) | 41/62 (66·1) |  |
| TB localization |  |  | >0·9 |
| Extrapulmonary only | 1/50 (2·0) | 2/74 (2·7) |  |
| Pulmonary | 46/50 (92·0) | 68/74 (91·9) |  |
| Pulmonary and extrapulmonary | 3/50 (6·0) | 4/74 (5·4) |  |
| Bilateral lung involvement | 25/43 (58·1) | 42/61 (68·9) | 0·3 |
| Any lung cavity | 38/49 (77·6) | 59/73 (80·8) | 0·7 |
| Smear grade |  |  | 0·7 |
| Negative | 11/47 (23·4) | 14/68 (20·6) |  |
| Positive 1+ | 11/47 (23·4) | 23/68 (33·8) |  |
| Positive 2+ | 9/47 (19·1) | 11/68 (16·2) |  |
| Positive 3+ | 12/47 (25·5) | 17/68 (25·0) |  |
| Positive 4+ | 4/47 (8·5) | 3/68 (4·4) |  |
| Previous anti-TB treatment history |  |  | 0·2 |
| None | 6/50 (12·0) | 17/70 (24·3) |  |
| First-line drugs | 3/50 (6·0) | 4/70 (5·7) |  |
| Second-line drugs | 41/50 (82·0) | 49/70 (70·0) |  |
| Any adverse events | 9/50 (18·0) | 26/74 (35·1) | 0·038 |
| ^1^Pearson's Chi-squared test; Fisher's exact test; Wilcoxon rank sum test.  Abbreviations: BDQ, bedaquiline; TB, tuberculosis; IQR, interquartile range.  ^a^Of the 104 patients treated with bedaquiline and with available treatment outcomes, 3 were excluded due to missing BDQ susceptibility results. | | | |

**Table S12.** Sensitivity analyses for the logistic regression.

| **Characteristic** | **Successful outcome** | **Unsuccessful outcome** | **Unadjusted OR (95% CI)** | **P-value** | **Adjusted OR (95% CI)** | **P-value** |
| --- | --- | --- | --- | --- | --- | --- |
| Main analysis | | | | | | |
| No. of effective drugs, median (IQR) | 6 (5, 8) | 6 (5, 8) | 0·8 (0·6, 1·0) | 0·06 | 0·7 (0·5, 0·9) | 0·006 |
| Sensitivity analysis 1: including “DST not available” as effective drugs. | | | | | | |
| No. of effective drugs, median (IQR) | 8 (6, 9) | 8 (7, 9) | 0·9 (0·8, 1·1) | 0·3 | 0·9 (0·7, 1·1) | 0·3 |
| Sensitivity analysis 2: considering drugs where DST was not available in >40% of patients as ineffective^1^. | | | | | | |
| No. of effective drugs, median (IQR) | 4 (3, 5) | 4 (3, 5) | 0·8 (0·7, 1·0) | 0·08 | 0·8 (0·6, 1·0) | 0·04 |

Abbreviations: OR, odds ratio; CI, confidence interval; IQR, inter quartile range; DST, drugs susceptibility testing.

^1^Includes kanamycin, cycloserine/terizidone, P-aminosaliscylic acid, pretomanid, carbapenem + amoxicillin/clavulanic acid.

**Table S13.** Sensitivity analysis for the Cox regression.

| **Characteristic** | **Total risk time (months)** | **Unsuccessful outcome** | **Unadjusted HR (95% CI)** | **P-value** | **Adjusted HR (95% CI)** | **P-value** |
| --- | --- | --- | --- | --- | --- | --- |
| Main analysis | | | | | | |
| No. of effective drugs, median (IQR) | 2708 | 101 | 0·9 (0·8, 1·1) | 0·2 | 0·9 (0·8, 1·1) | 0·2 |
| Sensitivity analysis 1: including “DST not available” as effective drugs. | | | | | | |
| No. of effective drugs, median (IQR) | 2708 | 101 | 1·1 (1·0, 1·2) | 0·06 | 1·1 (1·0, 1·2) | 0·06 |
| Sensitivity analysis 2: considering drugs where DST was not available in >40% of patients as ineffective^1^. | | | | | | |
| No. of effective drugs, median (IQR) | 2708 | 101 | 1·0 (0·9, 1·2) | 0·6 | 1·0 (0·9, 1·1) | 0·7 |

Abbreviations: HR, hazard ratio; CI, confidence interval; IQR, inter quartile range; DST, drugs susceptibility testing.

^1^Includes kanamycin, cycloserine/terizidone, P-aminosaliscylic acid, pretomanid, carbapenem + amoxicillin/clavulanic acid.

**Figure S1.** Observed versus predicted log-odds of unsuccessful treatment outcome by number of likely effective anti-tuberculous drugs.

**
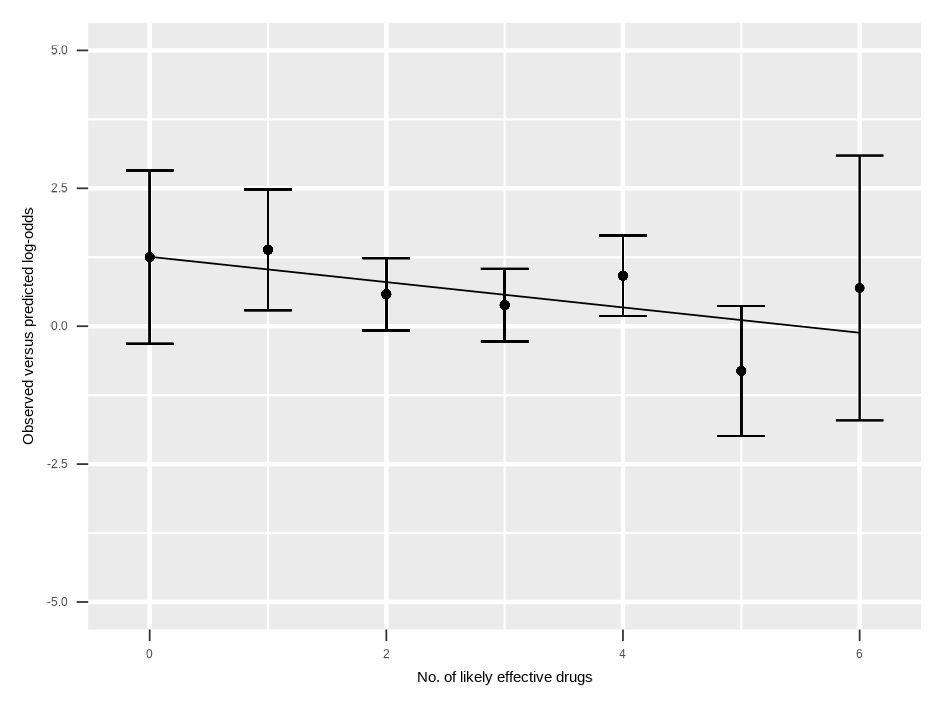
**

Points represent observed log-odds, and error bars indicate 95% confidence intervals. The line shows the fitted values from a logistic regression model treating drug count as a continuous variable. The approximately linear trend supports the assumption of linearity between drug count and log-odds in the model.

**Figure S2.** Observed versus predicted log-odds of unsuccessful treatment outcome by number of administered anti-tuberculosis drugs.


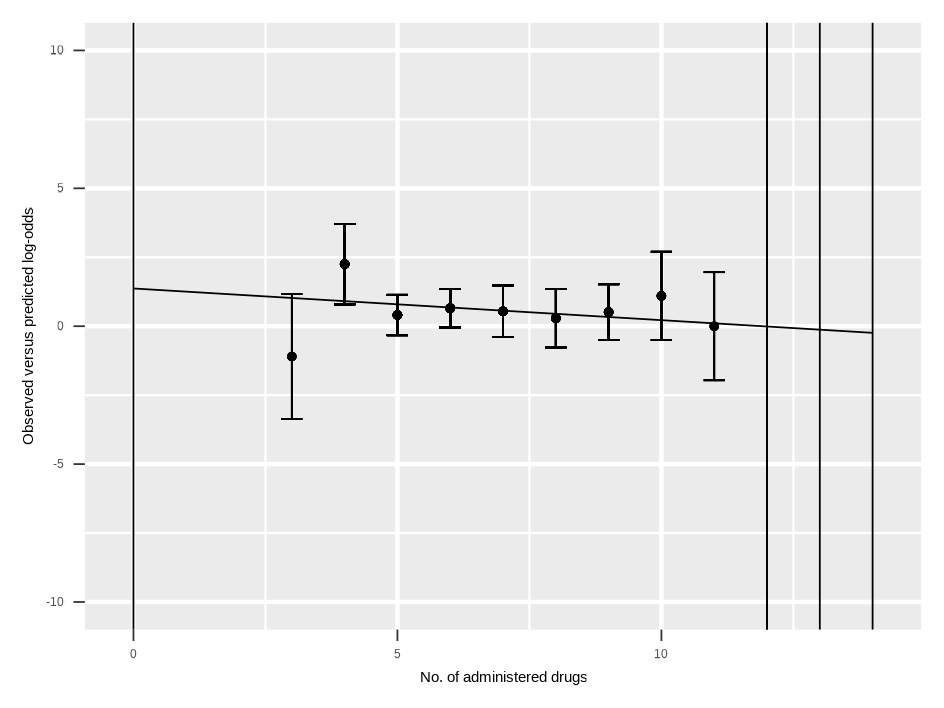


Points represent observed log-odds, and error bars indicate 95% confidence intervals. The line shows the fitted values from a logistic regression model treating drug count as a continuous variable. The approximately linear trend supports the assumption of linearity between drug count and log-odds in the model. Wider confidence intervals at extreme values reflect sparse data in those strata.

**Figure S3.** Univariable and multivariable Cox regression analysis of risk factors for unsuccessful treatment outcome in 154 extensively drug-resistant tuberculosis patients from 16 countries in the WHO European Region (2017-2023).

**
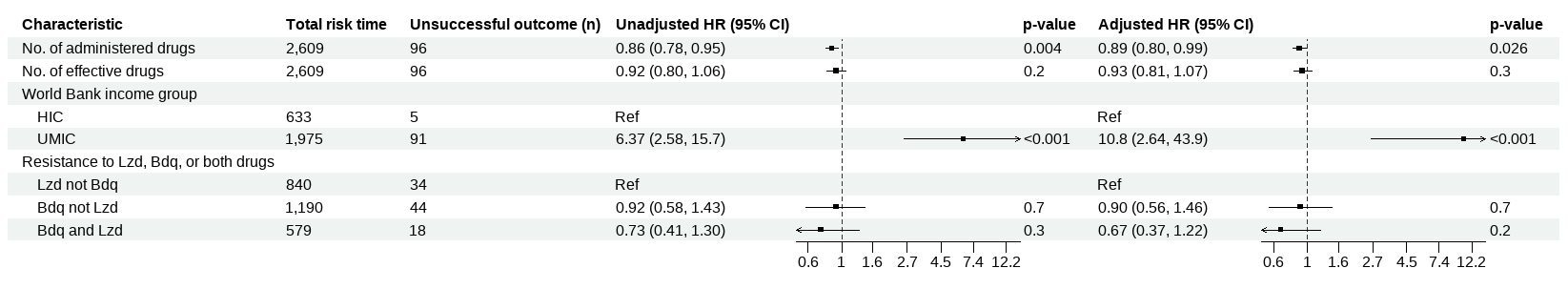
**

Schoenfeld residuals were used to test the proportional hazards assumption. In the multivariable Cox regression, the number of likely effective drugs reduced the hazard of unsuccessful outcomes (aHR: 0·9, 95% CI: 0·8, 1·1) although the association was not statistically significant (p = 0·2) (Figure 5B). Conversely, being treated in an UMIC significantly increased the hazard of unsuccessful outcomes (aHR: 10·8, 95% CI: 2·6, 43·9).

**Figure S4. Participating countries and the number of reported patients with extensively drug-resistant tuberculosis, by country.**

**
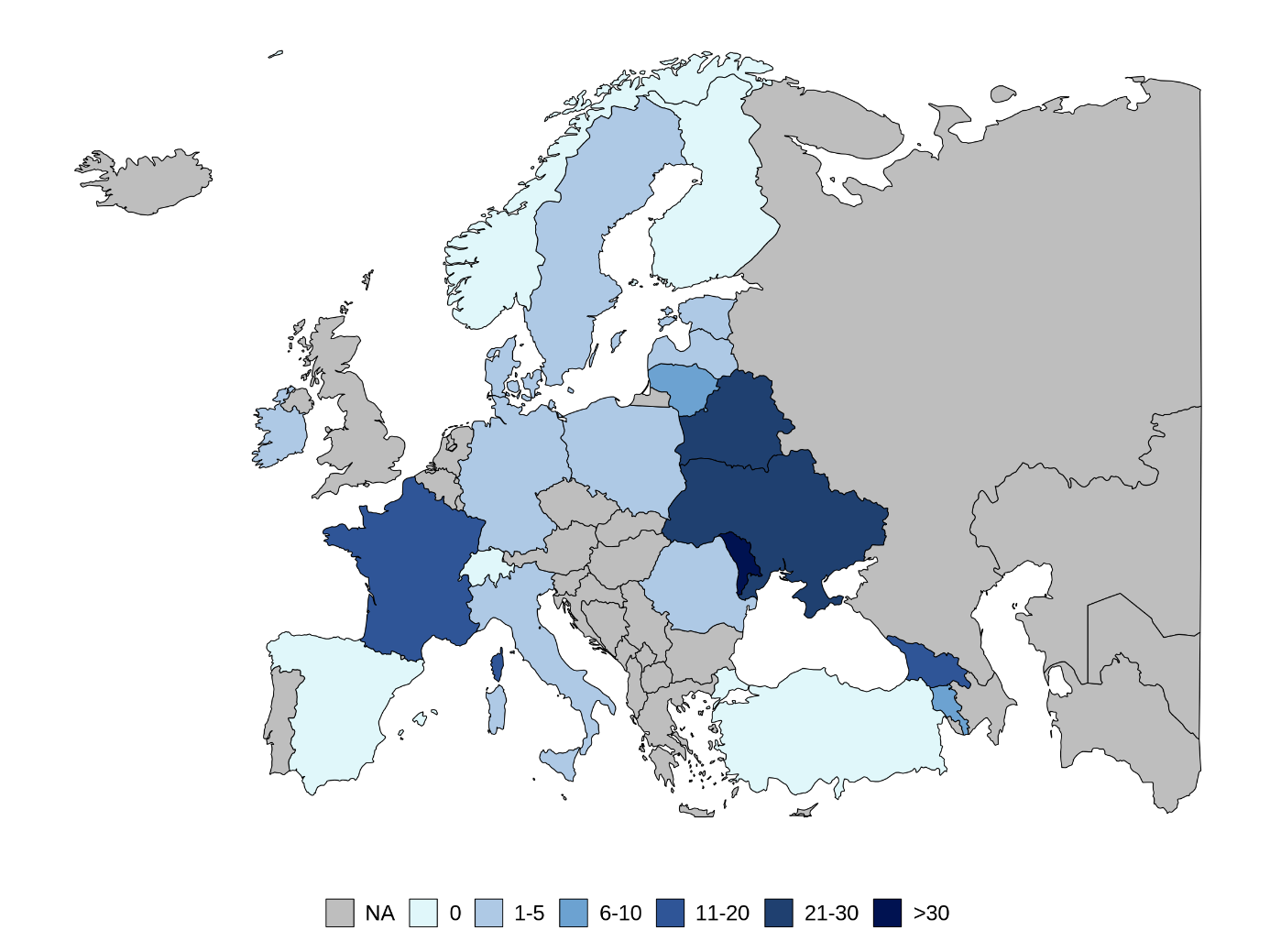
**

Fourty-two centres were invited to participate in the study. Twenty-four responded among which six had not treated any patients with extensively drug-resistant tuberculosis during the study period. Hence, 18 centres contributed data.

**Figure S5**. Overview of anti-tuberculosis drugs included in the regimen (A) and drug susceptibility testing results (composite genotypic/phenotypic) (B) of 188 extensively drug-resistant tuberculosis patients from 16 countries in WHO Europe Region (2017-2023).

**
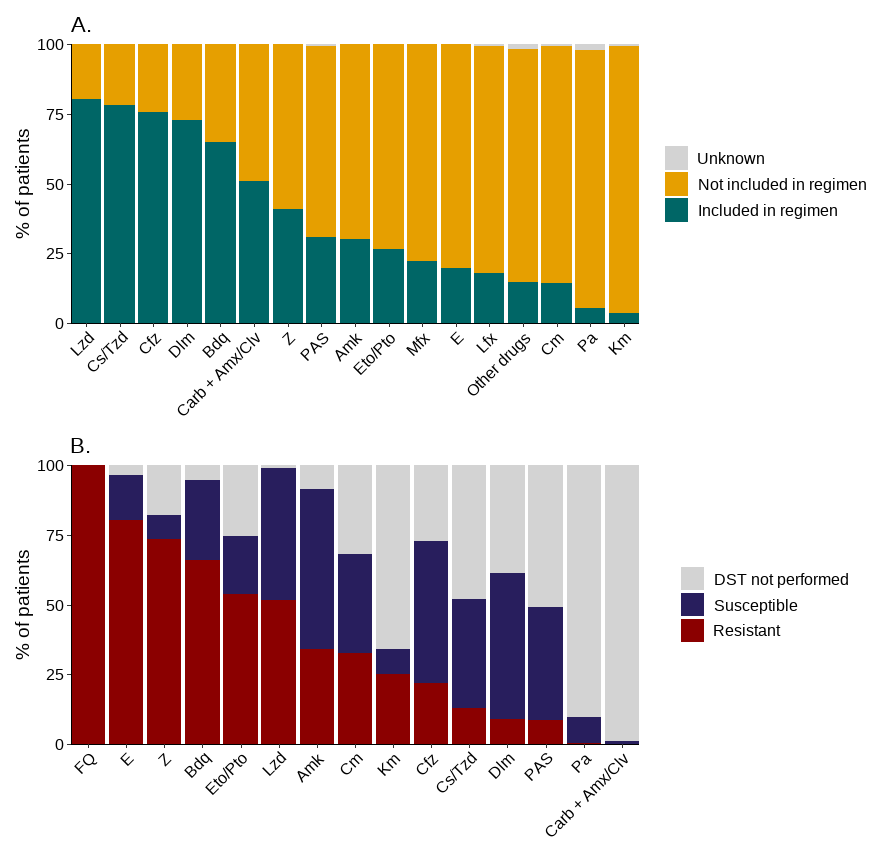
**

Abbreviations: Cfz, clofazimine; Cs/Tzd, cycloserine/terizidone; E, ethambutol; Dlm, delamanid; Pa, pretomanid; Z, pyrazinamide; Carb + Amx/Clav, carbapenem + amoxicillin/clavulanic acid; Amk, amikacin; Cm, capreomycin; Km, kanamycin; Eto/Pto, ethionamide/prothionamide; PAS, P-aminosaliscylic acid.

**Figure** **S6:** Crude comparison of treatment outcomes for patients with rifampicin-/multidrug-resistant, pre-extensively drug-resistant, and extensively drug-resistant tuberculosis patients from 16 countries in the WHO Europe Region (2017-2023).


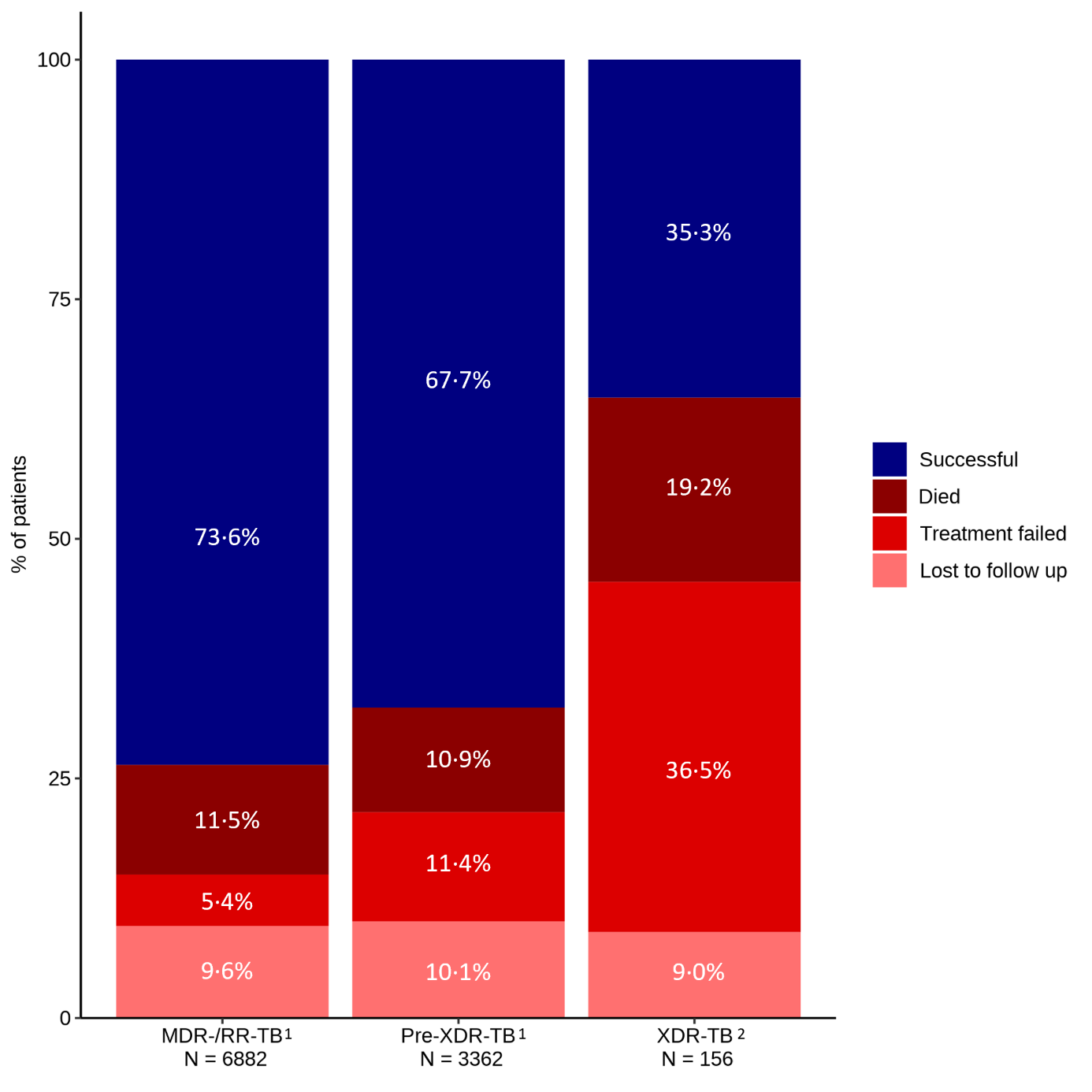


Abbreviations: MDR-/RR-TB, multidrug/rifampicin-resistant tuberculosis; Pre-XDR-TB, pre-extensively drug-resistant tuberculosis; XDR-TB, extensively drug-resistant tuberculosis.
^1^based on aggregate data from 12 out of 18 participating centres.
^2^based on patient-level data from the participating centre

**Figure S7:** Treatment success rates for MDR/RR-TB, pre-XDR-TB, and XDR-TB: random-effects meta-analysis by country.

**
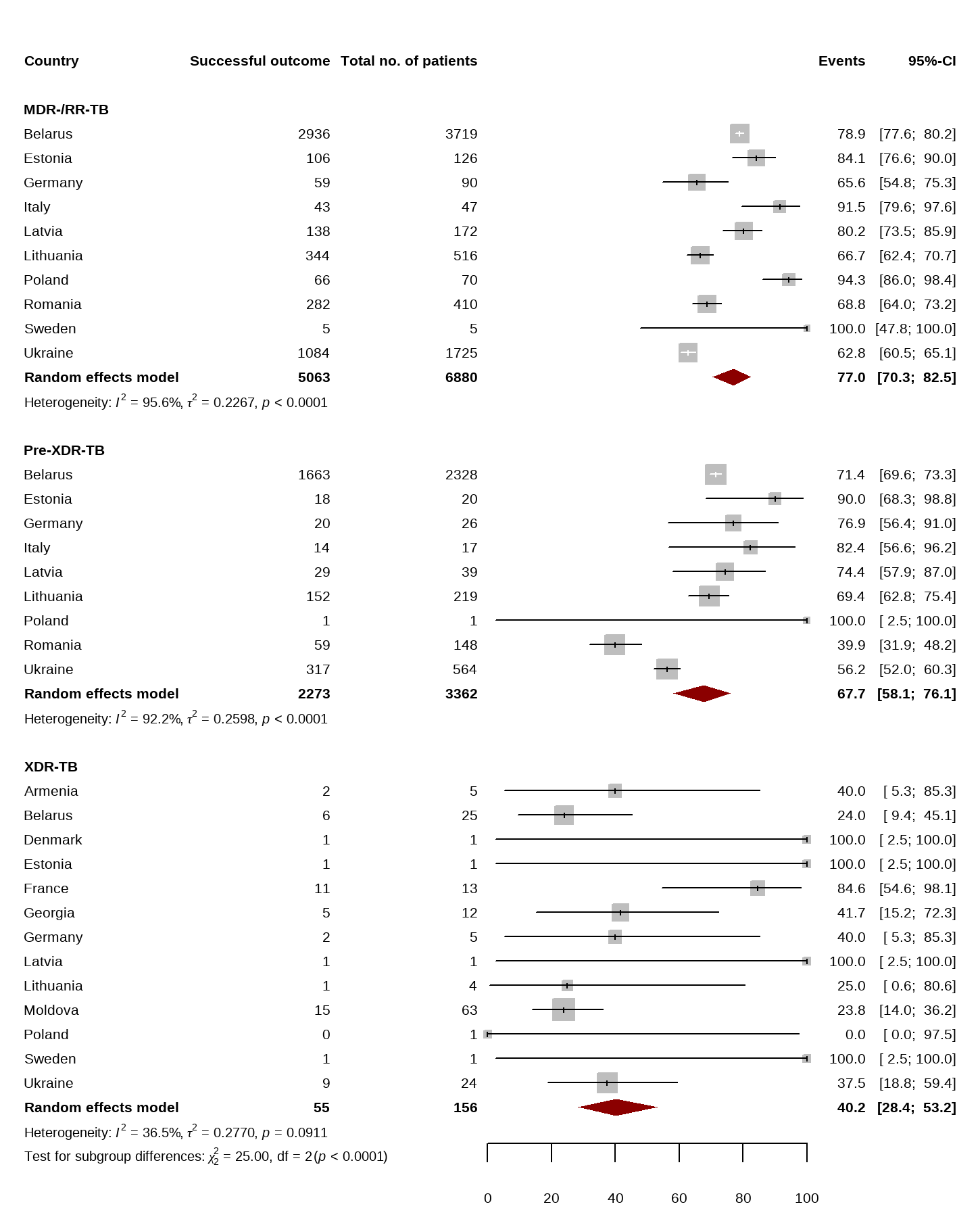
**

Abbreviations: MDR-/RR-TB, multidrug resistant/rifampicin resistant tuberculosis; pre-XDR-TB, pre-extensively drug-resistant tuberculosis; XDR-TB, extensively drug-resistant tuberculosis.

**Figure S8:** Pooled proportion of patients who died for MDR/RR-TB, pre-XDR-TB, and XDR-TB: random-effects meta-analysis by country.


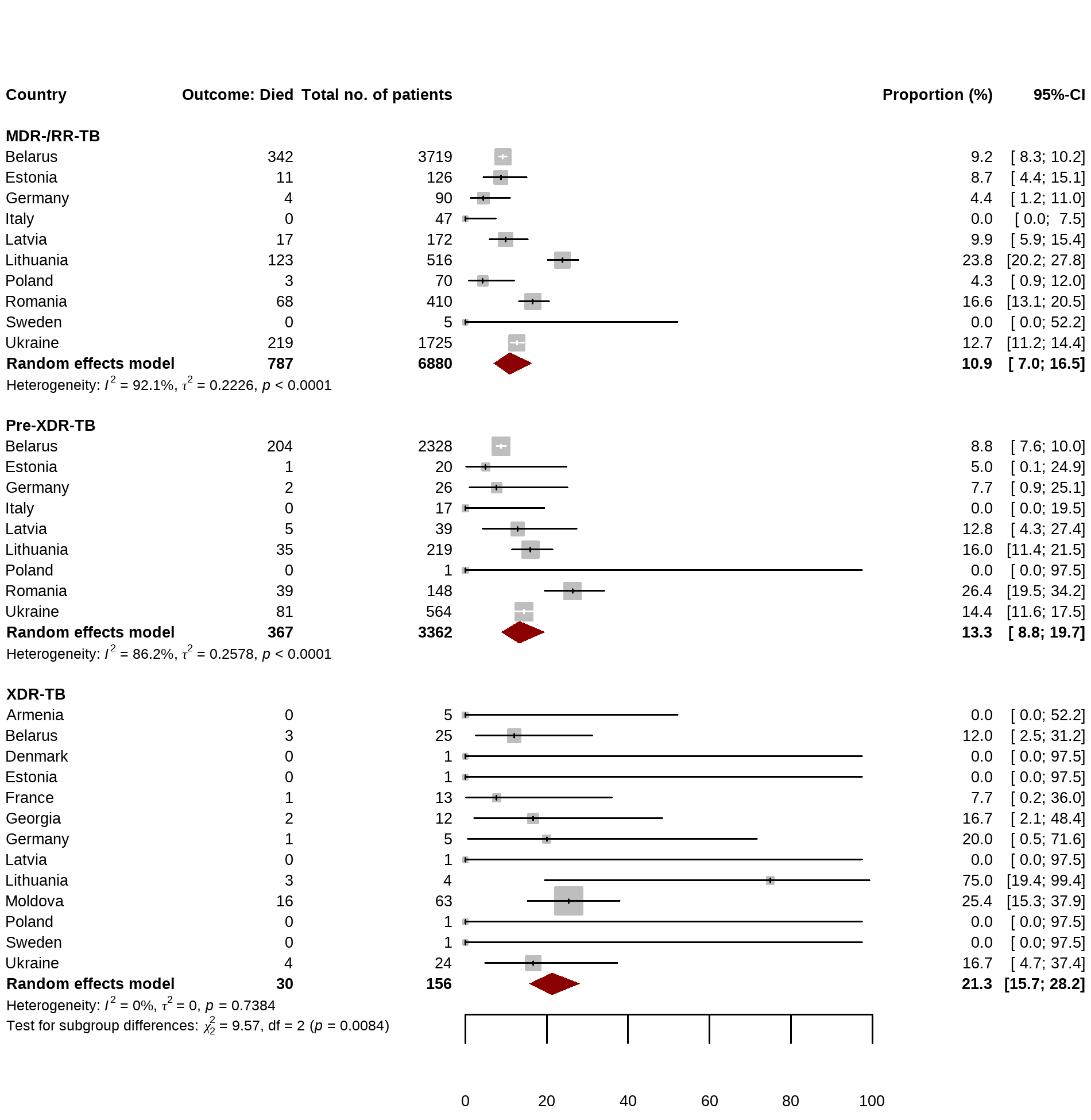


Abbreviations: MDR-/RR-TB, multidrug resistant/rifampicin resistant tuberculosis; pre-XDR-TB, pre-extensively drug-resistant tuberculosis; XDR-TB, extensively drug-resistant tuberculosis.

**Figure S9:** Pooled proportion of patients with treatment failure for MDR/RR-TB, pre-XDR-TB, and XDR-TB: random-effects meta-analysis by country.


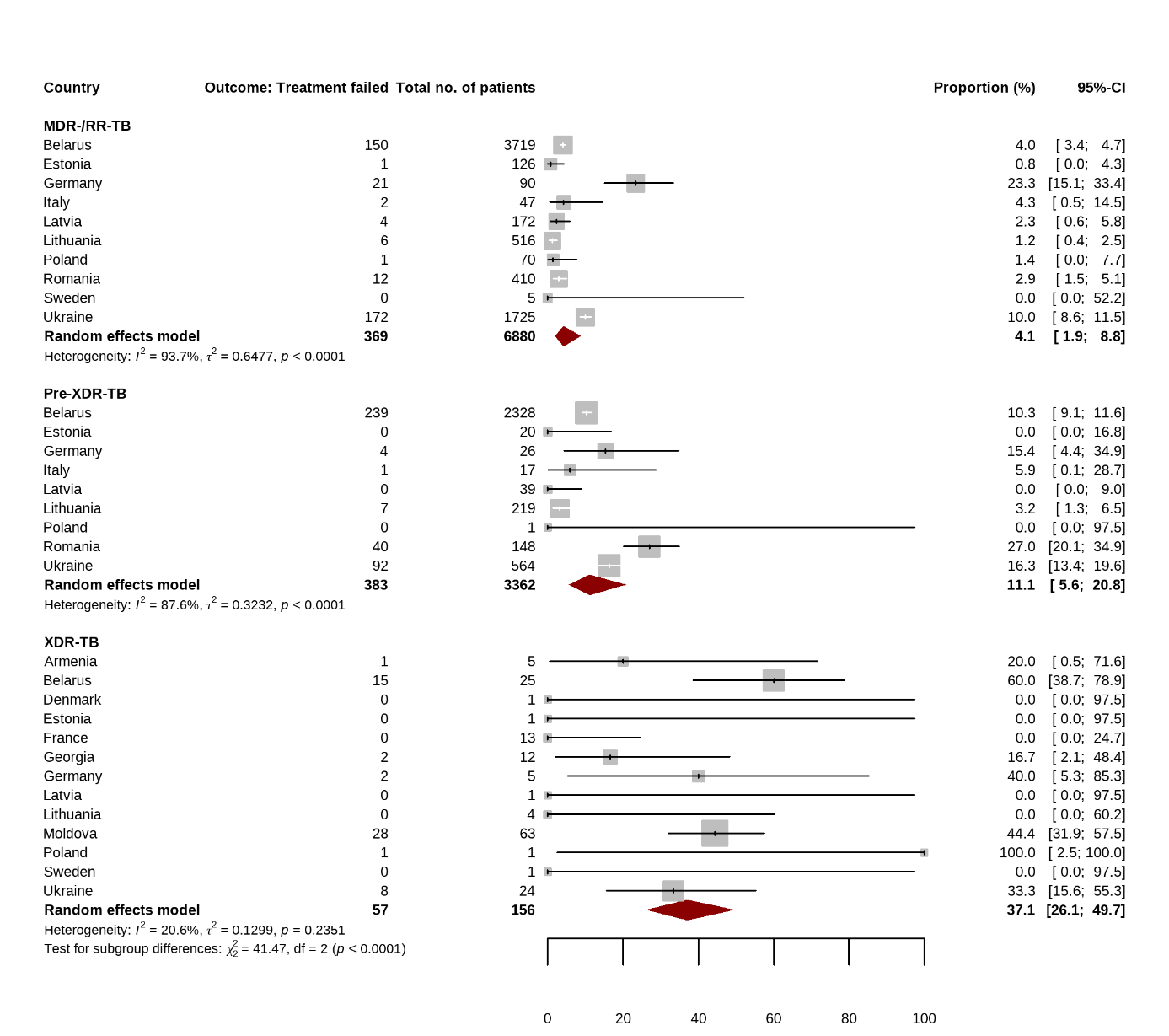


Abbreviations: MDR-/RR-TB, multidrug resistant/rifampicin resistant tuberculosis; pre-XDR-TB, pre-extensively drug-resistant tuberculosis; XDR-TB, extensively drug-resistant tuberculosis.

**Figure S10:** Pooled proportion of patients who were lost to follow-up for MDR/RR-TB, pre-XDR-TB, and XDR-TB: random-effects meta-analysis by country.


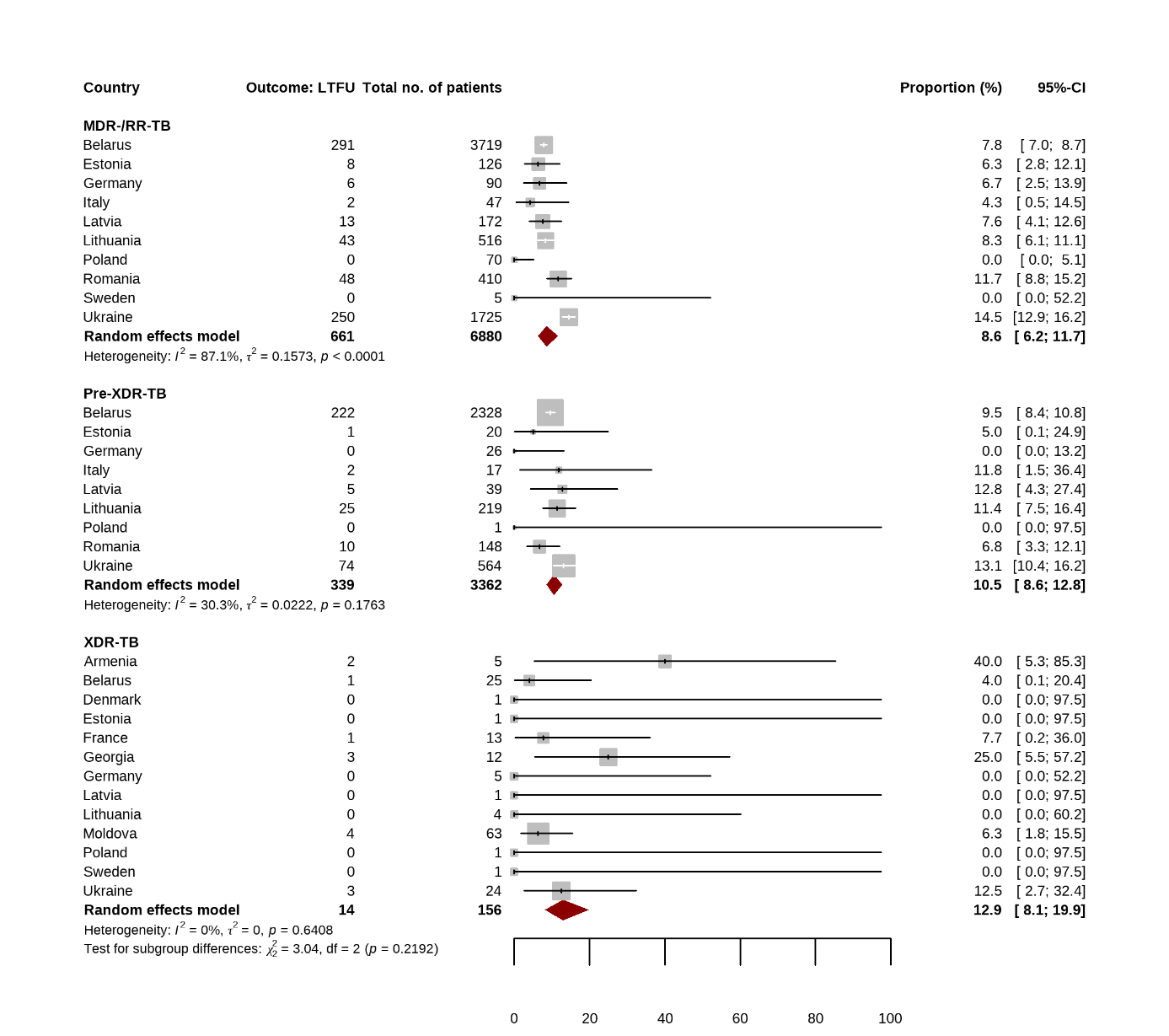


Abbreviations: LTFU, lost to follow-up; MDR-/RR-TB, multidrug resistant/rifampicin resistant tuberculosis; pre-XDR-TB, pre-extensively drug-resistant tuberculosis; XDR-TB, extensively drug-resistant tuberculosis.

**Figure S11.** Forest plot of descriptive analysis of extensively drug-resistant tuberculosis patients with evaluated outcomes (N = 156). The plot shows pooled percentages, including 95% confidence intervals with country-specific random effects, for each individual treatment outcome.


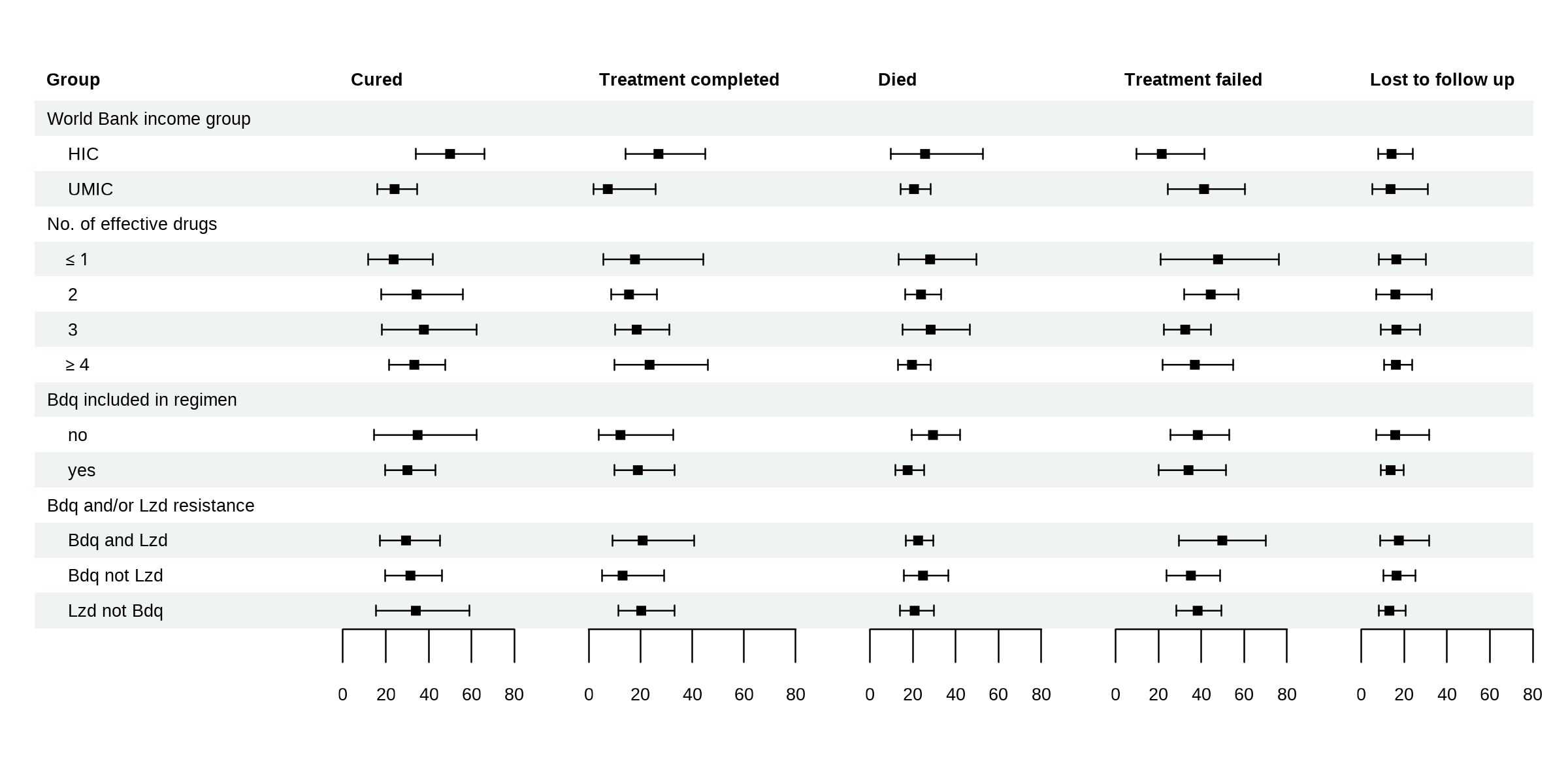


Abbreviations: HIC, high-income countries; UMIC, upper-middle-income country; Bdq, bedaquiline; Lzd, linezolid

**Figure S12:** Treatment success in XDR-TB patients with bedaquiline resistance who received BDQ versus other patients: random-effects meta-analysis by country.


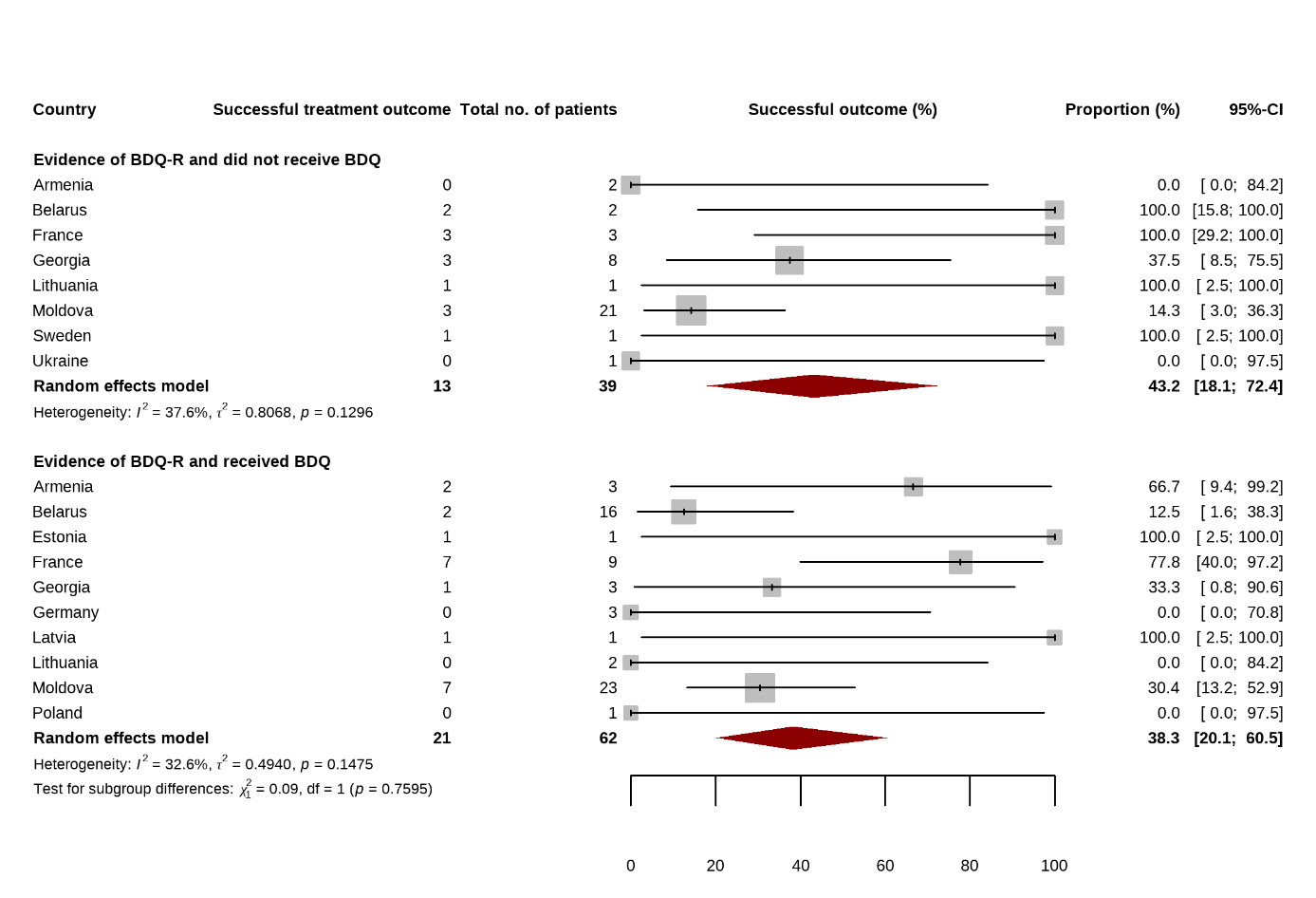


Abbreviations: BDQ-R, bedaquiline resistance; BDQ, bedaquiline.

**Figure S13.** Kaplan-Meier curves for time to unsuccessful outcomes and time to mortality for the whole cohort and subgroups (N = 154)


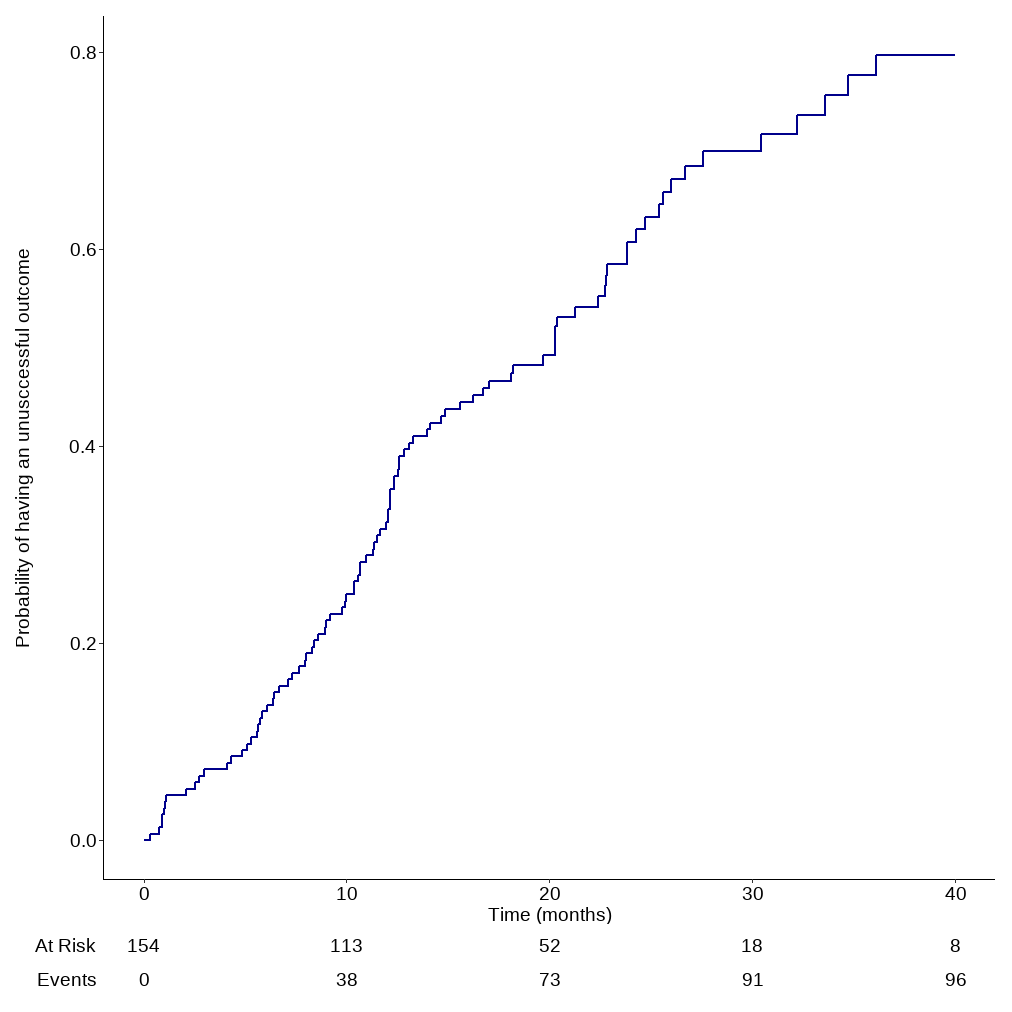

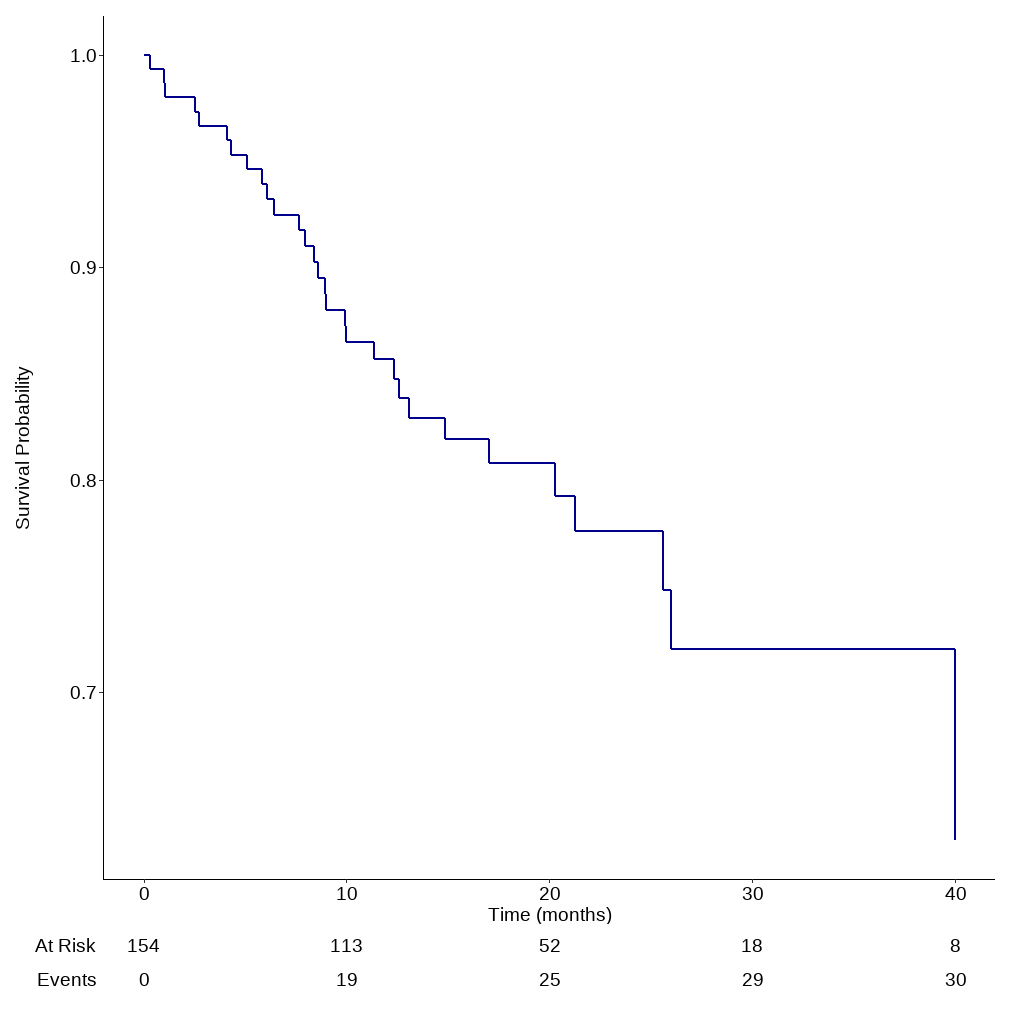

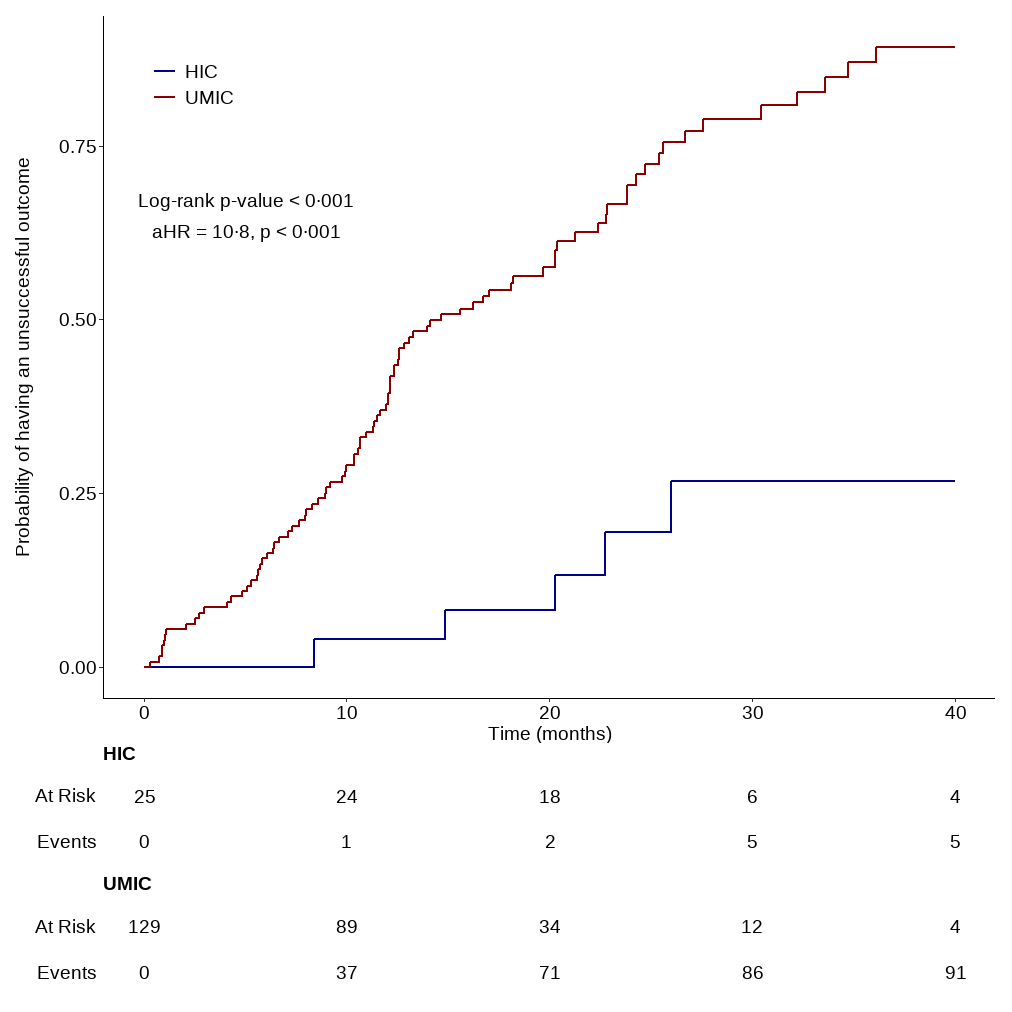

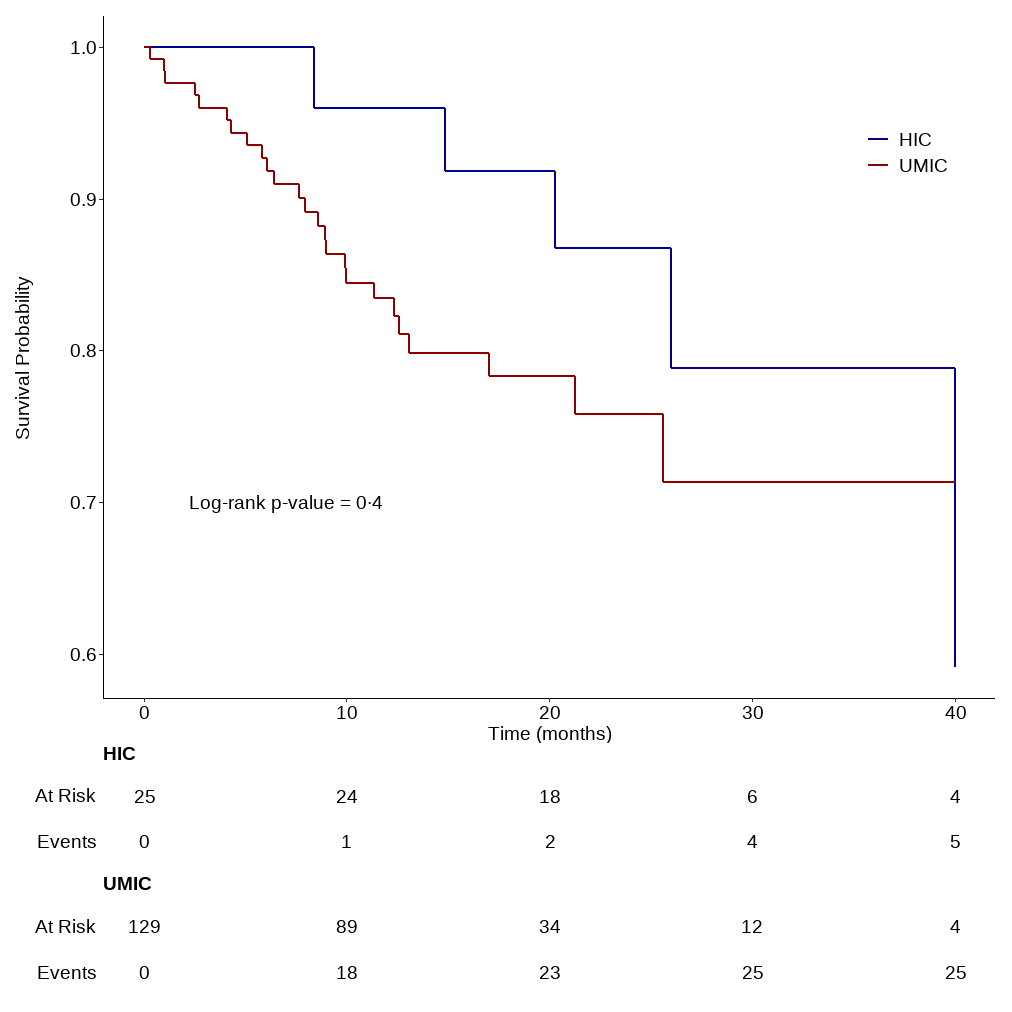


Abbreviations: HIC, high-income countries; UMIC, upper-middle-income countries
